# Large differences between UK black carbon emission factors

**DOI:** 10.1186/s13021-025-00306-3

**Published:** 2025-07-02

**Authors:** Adam Brighty, Iain Staffell, Helen ApSimon

**Affiliations:** Centre for Environmental Policy, Imperial College London, London, UK

## Abstract

**Introduction:**

Black carbon (BC) is a pollutant that illustrates strong links between climate warming and adverse health effects from air pollution. No standardised measurement technique for BC emissions has been implemented, making emissions and estimates highly uncertain. In this study, we evaluate two UK-based BC emission factor databases calculated using two distinct.

**Methods:**

the National Atmospheric Emissions Inventory (NAEI) and the Greenhouse Gas and Air Pollution Interactions and Synergies (GAINS) model database from IIASA. The scope of this investigation was limited to the 1 A (Fuel Consumption) NFR code, which comprised the largest BC-emitting activities in the UK. Comparisons were made between a reference NAEI value and a range of low (e.g., highest abatement, newest technology), medium, and high GAINS emission factors. The NAEI value sat outside the GAINS BC ranges across 64% of the selected 1 A sources, most evidently within industrial combustion. By comparison, PM_2.5_ and NO_x_ emission factors within the same databases showed less frequent disagreement, with 26% and 46%, respectively, of the GAINS sources not overlapping with the NAEI reference. A complementary BC emissions estimate, using NAEI activity data, found the highest variance in emissions to be within industrial, domestic, and agricultural combustion sources. Overall, this paper highlights the need to understand the differences behind these BC emission factors and to bring them into closer alignment.

**Supplementary Information:**

The online version contains supplementary material available at 10.1186/s13021-025-00306-3.

## Introduction

### Background

Black carbon (BC) is “carbonaceous particulate matter that absorbs light” and is emitted during the incomplete combustion of carbonaceous fuels, such as biomass and fossil fuels (e.g., natural gas and oil) [1, 2]. A pollutant that illustrates climate warming capabilities, BC strongly absorbs infrared radiation, changes the optical properties of clouds, and reduces the planetary albedo when deposited onto snow and ice [2]. The reduction in albedo is most keenly felt within glacial locations, such as the Arctic, Andes, Himalayas, and Alps, leading to more rapid warming, ice melting, and sea level rise [3, 4]. The summation of these effects leads to a net positive planetary warming effect [2]. Given the short atmospheric lifetime, the removal of BC emission sources is followed quickly by reductions in BC concentrations and deposition [5]. The most notable emissions sources include forest fires and anthropogenic sources such as agricultural waste burning, transport and domestic cooking and heating [2]. However, the increased use of plant and wood biomass as a renewable source for power generation in Europe is expected to increase the BC contribution from biomass combustion in the future [6].

BC is also known to be an atmospheric pollutant with deleterious human health effects. BC typically resides within PM_2.5_, which is known to penetrate the respiratory system and has been causally linked to mortality effects and severe diseases [7, 8]. BC particles have also shown their proficiency for allowing gas-phase organic compounds to coat the surface of the particle, causing BC to become more toxic with ageing [9]. Oxley et al. completed an investigation looking at the impact of BC emissions in London across a range of different BC toxicity levels, derived from epidemiological studies [10]. They found that, for both high and low BC toxicities, reductions in BC emissions from road transport would provide the largest improvement in public health in London over other urban sectors, such as domestic and public combustion.

There are clear arguments to reduce BC emissions on both health and environmental grounds. Efforts to minimise these emissions fall under the Convention on Long-Range Transboundary Air Pollution. Historically, the convention has focused predominantly on PM_2.5_ by mass for controlling particulate pollution. As a result, short-lived climate forcers, such as BC and methane, have tended to fall between air quality and climate discussions without being the focus of either area. This has been recognised by policymakers in recent years, with last year’s UNECE Saltsjöbaden VII workshop stating “…the effects of air pollution mitigation on climate forcing should be considered during health and ecosystem impact assessments, and in the development of emission mitigation policies” [11]. BC has also been added to the list of pollutants under consideration in the renegotiation of the Gothenburg Protocol, and other organisations have also recognised its importance. For example, the WHO’s global air quality guidelines encourage governments to begin taking ambient BC measurements, producing BC inventories, reducing BC emissions, and removing BC sources [12]. The UK utilises the National Emissions Ceiling Regulations 2018, which are based on the EU National Emissions Ceiling Directive 2016/2284/EU, as a legal framework to achieve emission reductions. These regulations establish emission reduction targets for five primary air pollutants for both 2020 and 2030, with BC’s submission being voluntary [13]. To effectively address BC abatement, it is essential that countries have a reliable emissions inventory and source apportionment, identifying the most critical BC sources, and understand how abatement measures will impact emissions. This requires a well-defined emission factor (EF) for the relevant source and pollutant, which has not historically been the case for black carbon.

### Black carbon emission factor methods

Unlike other atmospheric pollutants, there is no official measurement standard for BC emissions. This fundamental problem hinders the development of BC measurement techniques. Some approaches to BC measurements use elemental carbon as a surrogate, owing to its light-absorbing properties. This lack of a defined and standardised emissions test method has led to a large uncertainty remaining around the characterisation of BC emission factors and has limited improvements to EF databases. For example, from a UK perspective, annual BC emissions have an uncertainty range of between − 20% and + 50% [14].

**Fig. 1:**
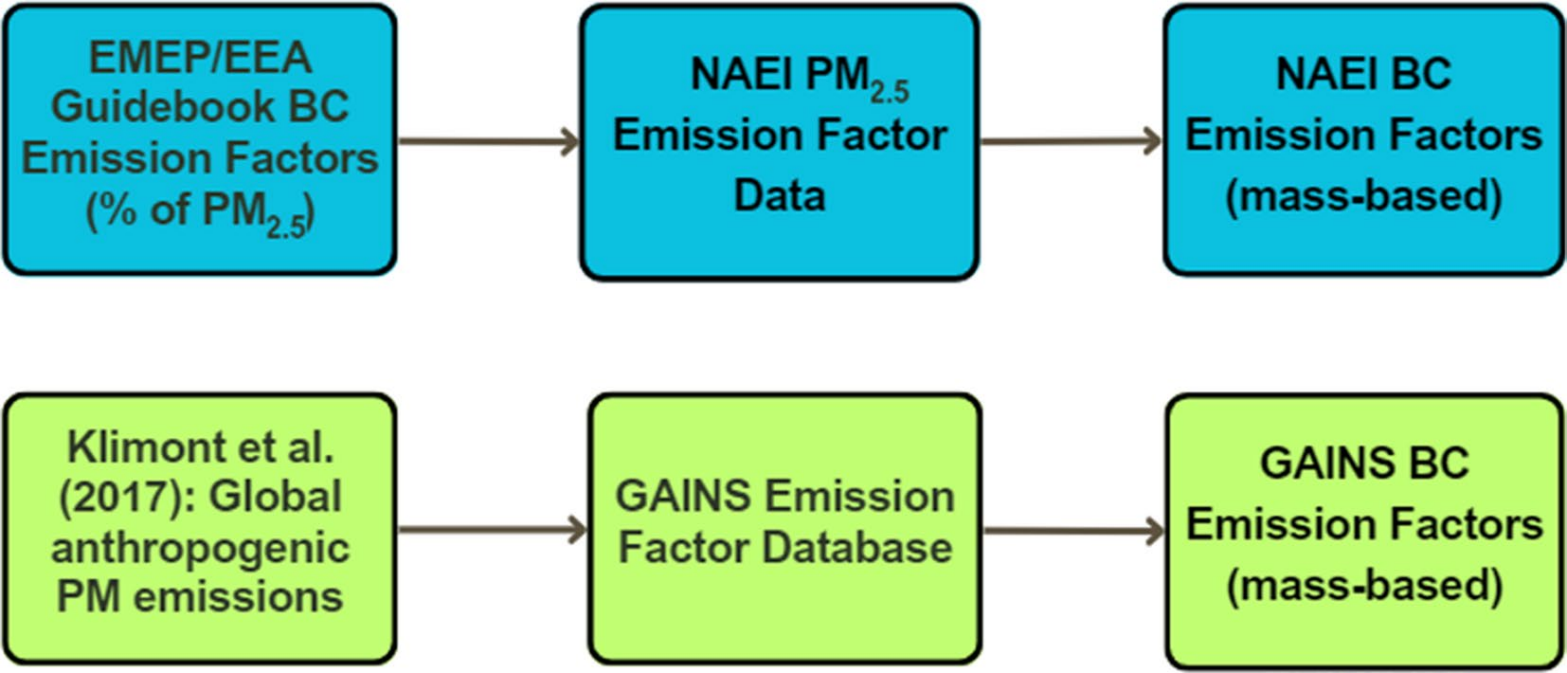
The differences in the ways the NAEI (top) and GAINS (bottom) BC EFs are created

Looking globally, BC emissions projections have been commonly completed by applying a percentage fraction of BC (%BC) to mass-based PM_2.5_ emission factors. This is the case within the US government’s SPECIATE database and, to a lesser extent, the European EMEP/EEA emissions inventory guidebook [13, 15]. The EMEP/EEA guidebook has begun the process of moving away from the %BC method, but the current database still retains a large proportion of emission factors using this technique. The use of guidebook values is recommended for each European Member State, including the UK, where the National Atmospheric Emissions Inventory (NAEI) completes emissions estimates on behalf of the UK. However, the NAEI retain the right to move away from the stock EMEP/EEA values when they have sufficient data for a given source. This may be necessary, given the less granular European-wide emission factors included in the EMEP/EEA Guidebook. The Guidebook’s emission factors for PM_2.5_ and the %BC fraction represent an accumulation of expert and stakeholder consultation, existing policy emission limit values, and previous literature sources [13]. As the original EMEP/EEA Guidebook intends, the NAEI do not present BC EFs as a percentage of PM_2.5_ values. To aid simplicity within the NAEI database, the mass-based BC EF is derived using the %BC fraction and PM_2.5_ EFs before its inclusion in the NAEI database. The NAEI typically present their BC EFs as single values, which are aggregated across a range of technologies. This has been summarised in Fig. 1.

The method used by the NAEI depends on the initial PM_2.5_ EF and %BC factor:

1 $$\:{Emissions}_{BC}=(Activity\:Data\times\:{EF}_{{PM}_{2.5}})\:\times\:{Ratio}_{{BC\:\::\:PM}_{2.5}}$$

This dependency on both the PM_2.5_ EF and %BC value can compound uncertainties and inaccuracies in both key factors. As an example, in the UK, the emissions limit for total filterable particulate matter within ‘other industrial combustion’ of biomass is set at 30 g/GJ (or 108 g/MWh), in line with the Renewable Heat Incentive [16]. The PM_2.5_ EF, a smaller fraction within total filterable PM, used by the NAEI for ‘other industrial combustion’ of biomass, is significantly higher than the Renewable Heat Incentive value, at 484 g/MWh. Using the %BC technique here would therefore predict BC emissions from other industrial combustion to be over 4 times higher than allowed by the Renewable Heat Incentive.

The %BC method also assumes that changes in PM_2.5_ emissions will yield an equivalent change in BC emissions. However, emissions of PM_2.5_ and elemental carbon (assumed to be equivalent to BC) were shown to correlate poorly with one another under changing combustion conditions within residential wood burning; elemental carbon emissions were much less susceptible to changes in conditions (e.g., wood moisture content and fuel load within the stove) than PM_2.5_ [17].

Moving away from BC EFs based on a fraction of PM_2.5_ to specific mass-based EFs (e.g., gBC/kWh) could help represent BC emissions more accurately. This means that BC emissions would no longer be reliant on both an accurate PM_2.5_ EF and %BC fraction, as is the case with the EMEP/EEA and NAEI method. Emissions would then be calculated more easily and directly:

2 $$\:{Emissions}_{BC}=Activity\:Data\times\:\:{EF}_{BC}$$

However, the aforementioned lack of a standardised BC measurement technique contributes to a high level of uncertainty around mass-based BC emission factors. As a result, EF databases with mass-based BC values are rare or only give partial coverage in databases, such as the EMEP/EEA guidebook [13].

One such example of a mass-based BC EF dataset is provided by Klimont et al. [18]. The authors utilised the GAINS model, combined with BC literature, measurement data, and policy implementations, to produce the first estimate of global anthropogenic BC emissions. A full subsequent BC EF inventory was included within the ECLIPSE (Evaluating the Climate and Air Quality Impacts of Short-Lived Pollutants) dataset at the end of the project. The GAINS model is used for the United Nations Economic Commission for Europe in their Centre for Integrated Assessment Modelling, and the country-specific mass-based BC dataset is included in the database on the GAINS model website. Like the NAEI and EMEP/EEA database, these emission factors are included within the ‘Western Europe’ region of the emission factor dataset and assumed to be relevant for the UK. The GAINS model distinguishes between a range of technologies for the same source, such as domestic heating within stoves, boilers, and fireplaces. It should be noted that the BC EFs for abated technologies were calculated based on percentage reductions from unabated technologies, which may be a limitation of the GAINS EF dataset. To our knowledge, GAINS has the only comprehensive mass-based BC EF database for UK emission sources.

Here, we compare the two different approaches to producing black carbon EFs for UK sources. This is the first analysis of its kind, where we assess the agreement between GAINS BC EFs, using a mass-based approach, and the EFs presented within the NAEI BC emissions inventory, which are calculated from a % of PM_2.5_. This analysis is not a direct critique of the two individual databases, given the inherent uncertainty surrounding BC measurements, nor does it indicate a preference for one method over another. This analysis examines the similarities and differences between the two methods.

## Method

**Fig. 2:**
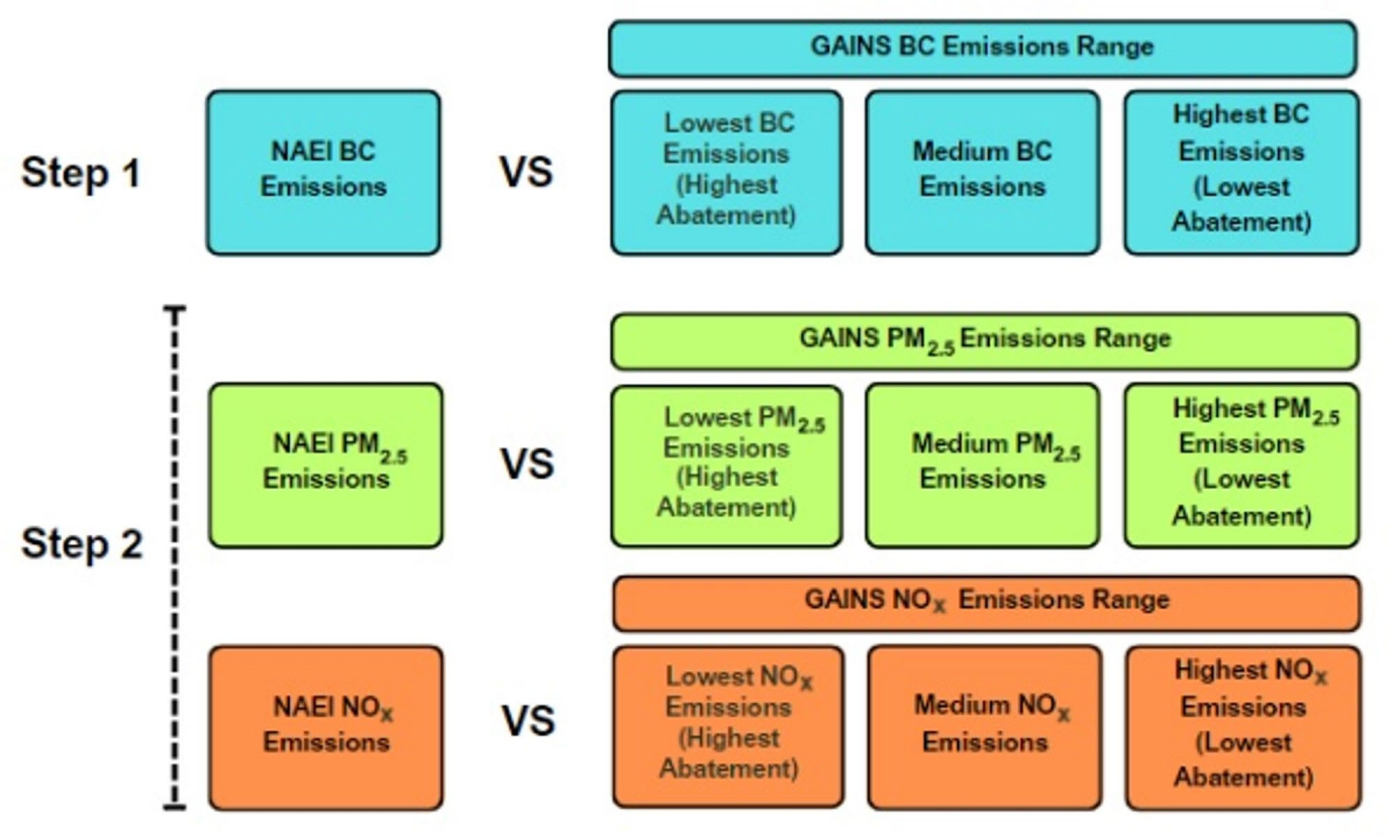
Methodology for the comparison between NAEI and GAINS BC, PM_2.5_, and NO_x_ emission factors

Figure 2 summarises the comparison method used within this study. To understand how GAINS BC EFs compare with NAEI values, where possible, low, medium, and high EFs were taken from the GAINS database. These values differed either in their levels of abatement, commonly within industrial combustion and electricity production, or in the range of technologies used for a given fuel, which was more common for domestic combustion sources. Overall, this provided three sets of GAINS BC EFs. By contrast, the NAEI typically provide a *singular* emission factor, aggregated across a range of technologies. To provide additional context, both PM_2.5_ and NO_x_ EFs were also evaluated across each database. These pollutants are more widely investigated than BC, with specific measurement standards in place. PM_2.5_ EFs are also important in defining BC EFs in the NAEI database. All emission factors investigated are listed in the supplementary information (SI) (Tables S1– S6). A complementary study comparing GAINS BC emissions estimates for 2021 with the NAEI estimates was also completed.

### Emission factor source selection

To assess which sources and sectors are most important for overall UK BC emissions, emissions estimates from the NAEI [14], across the period from 2015 to 2021 were evaluated. The NAEI disaggregates UK emissions sources using the standardised “Nomenclature for Reporting” (NFR) code format, agreed upon within the Convention on Long-Range Transboundary Air Pollution. This standardised format, with sources grouped into similar activities (or NFR codes), provides a consistent platform for countries’ submissions to air pollution conventions. Table 1 illustrates which sources are included in each 1 A NFR Code.

**Table 1:** Description of sources within 1 A NFR codes

NFR Code	Source Description
1 A	**Fuel Combustion Activities**
1A1	Public electricity and heat production, refineries, solid fuel manufacture
1A2	Combustion in manufacturing industries and construction
1A3	Aviation, road transport, shipping
1A4	Commercial and residential combustion, agriculture combustion, fishing
1A5	Other mobile combustion (recreational boats, military)

**Fig. 3:**
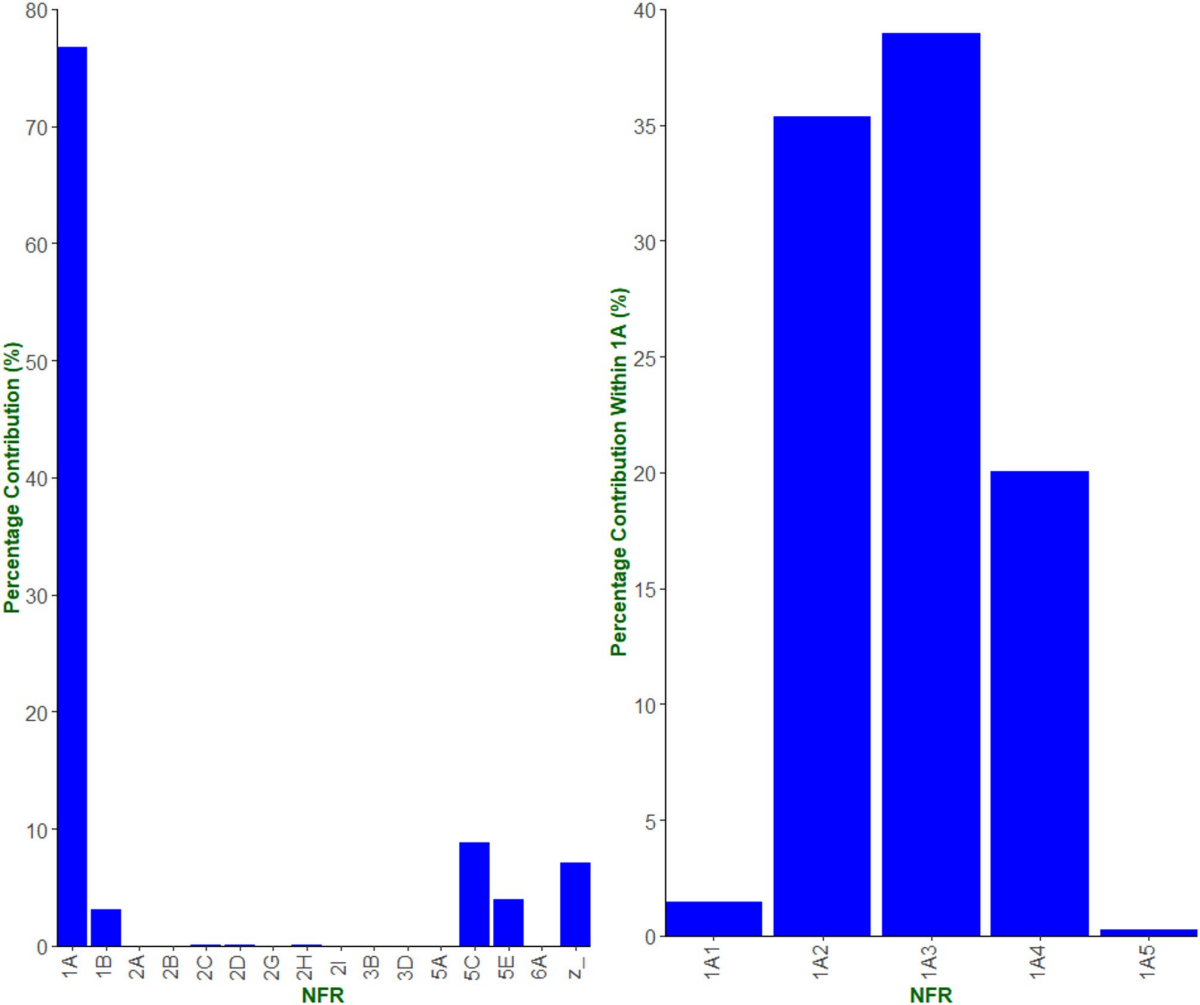
Left– annual average contribution to BC emissions from 2015–2021, according to the NAEI. Right– contribution in BC emissions from 1 A sub-sectors within 1 A, according to the NAEI

Figure 3 (left) illustrates the dominance of the NFR code 1 A (fuel combustion industries) activities in total BC emissions, contributing an annual average of around 77% of all UK BC emissions from 2015 to 2021. The remaining contribution to BC emissions came from NFR codes 5 C (waste incineration), 5E (other waste), and 1B (fugitive emissions from fuels). As a result, the study was restricted to solely 1 A emissions sources. To limit the scope further, sources that gave an average annual contribution to total BC emissions of less than 0.1% (to 2 d.p.) were omitted, leaving a total of 83 sources. The included 1 A emission sources in this study contributed an average of 74% to the total BC emissions from 2015 to 2021, according to the NAEI [14].

Across the 83 largest BC sources within 1 A, illustrated in Fig. S1 (SI), 3 came from 1A1 (energy industries), 17 from 1A2 (manufacturing industries and construction), 41 from 1A3 (transport), 21 from 1A4 (other sectors e.g., public and domestic combustion), and 1 from 1A5 (other– e.g., other mobile combustion). Figure 3 (right) illustrates the largest contributions from within 1 A came from 1A2 and 1A3. Other industrial combustion of biomass (within 1A2) contributed over 11% to total 1 A BC emissions.

### Emission factor comparison method

**Fig. 4:**
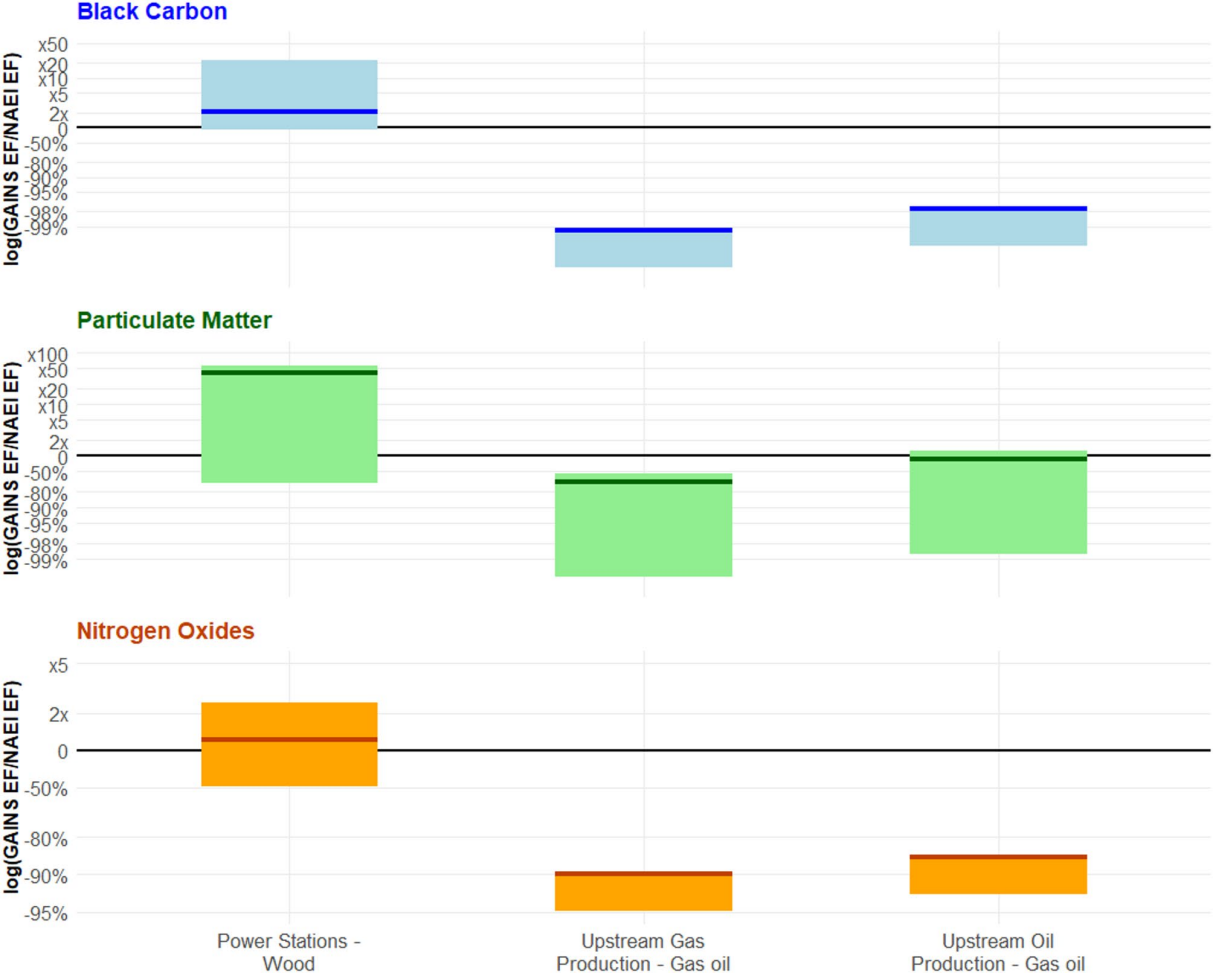
Ratio of GAINS/NAEI emissions factors (EFs), represented on a logarithmic axis, for selected sources within 1A1 for black carbon (BC), particulate matter (PM_2.5_), and nitrogen oxides (NO_x_). The y-axis indicates the difference in magnitude between the low, medium, and high GAINS emission factors and the NAEI emission factor. The upper and lower ends of each bar indicate the ratio of the GAINS/NAEI value when applying the highest and lowest GAINS EFs, respectively. The thick coloured line indicates the middle GAINS emission factor compared to the NAEI value. In some cases, the middle and high GAINS emission factors are the same. The black line at y = 0 indicates the point at which the GAINS and NAEI emission factors are equal. For the sources within 1A1, overlap of emission factors is seen in all cases for wood combustion in power stations, and for PM_2.5_ emission factors for gas oil combustion in upstream oil production. For the sources with no overlap, both PM_2.5_ and NO_x_ emission factors are closer to the reference NAEI value

**Fig. 5:**
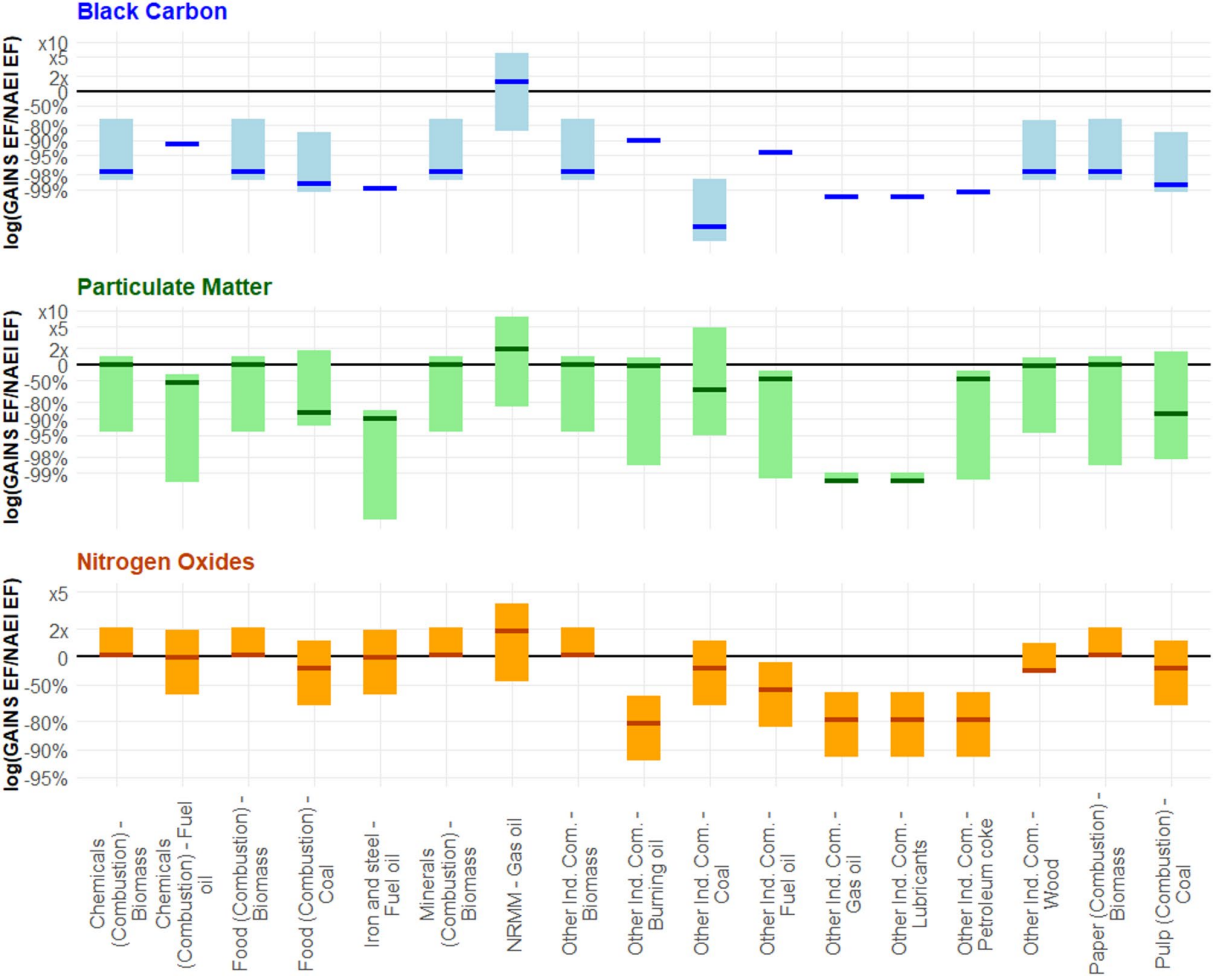
Ratio of GAINS/NAEI emissions factors (EFs), represented on a logarithmic axis, for selected sources within 1A2 for black carbon (BC), particulate matter (PM_2.5_), and nitrogen oxides (NO_x_). The y-axis indicates the difference in magnitude between the low, medium, and high GAINS emission factors and the NAEI emission factor. The upper and lower ends of each bar indicate the ratio of the GAINS/NAEI value when applying the highest and lowest GAINS EFs, respectively. The thick coloured line indicates the middle GAINS emission factor compared to the NAEI value. In some cases, the middle and low GAINS emission factors are the same, or only one value is provided– resulting in a single line with no bar. The black line at y = 0 indicates the point at which the GAINS and NAEI emission factors are equal. For the sources within 1A2, only one set of GAINS BC emission factors overlaps with the NAEI, whereas for both PM_2.5_ and NO_x_ 11 and 12 emission factor sets overlap with the NAEI value, respectively, with other sources also having closer agreement than for the BC equivalents. There is also much better agreement when looking at “other industrial combustion” of biomass, a dominant source, for PM_2.5_ and NO_x_ EFs than for the BC equivalents. NB: NRMM– Non-Road Mobile Machinery, Other Ind. Com.– Other industrial combustion

**Fig. 6:**
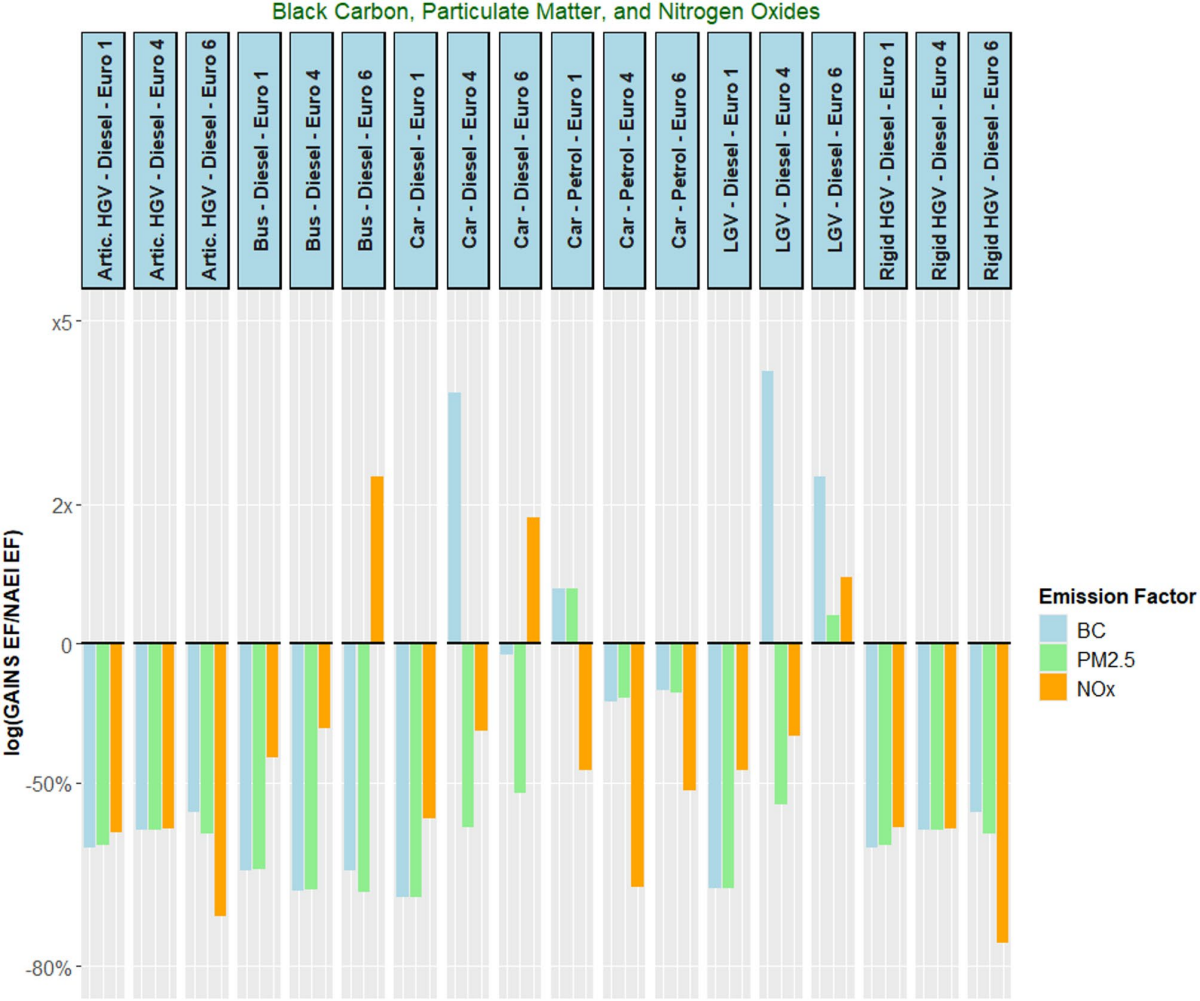
Ratio of GAINS/NAEI emissions factors (EFs), represented on a logarithmic axis, for selected exhaust-based road transport sources within 1A3 for black carbon (BC), particulate matter (PM_2.5_), and nitrogen oxides (NO_x_). These sources are selected based on fleet-aggregated BC emissions for different fuel and vehicle types (with no Euro-specific split). To allow for a like-for-like comparison, the Euro-specific GAINS exhaust EFs are compared to the EEA Guidebook Euro-specific EFs, which are used by the NAEI. The y-axis indicates the difference in magnitude between the GAINS emission factor and the NAEI emission factor. The black line at y = 0 indicates the point at which the GAINS and NAEI emission factors are equal. For the exhaust-based sources within 1A3 (excluding cold starts), BC and PM_2.5_ EFs in the NAEI and GAINS databases show very similar comparisons for most sources, with the exceptions being Euro 4 and Euro 6 cars and light goods vehicles. GAINS NO_x_ EFs are closer in magnitude to the NAEI value in 11 of the 18 sources than for the BC and PM_2.5_ equivalents, but disagreement is seen on Euro 6 heavy goods vehicles. Overall, across the 54 different comparisons (18 sources, 3 pollutants each), only 9 GAINS EFs were found to be larger than the corresponding NAEI value

**Fig. 7:**
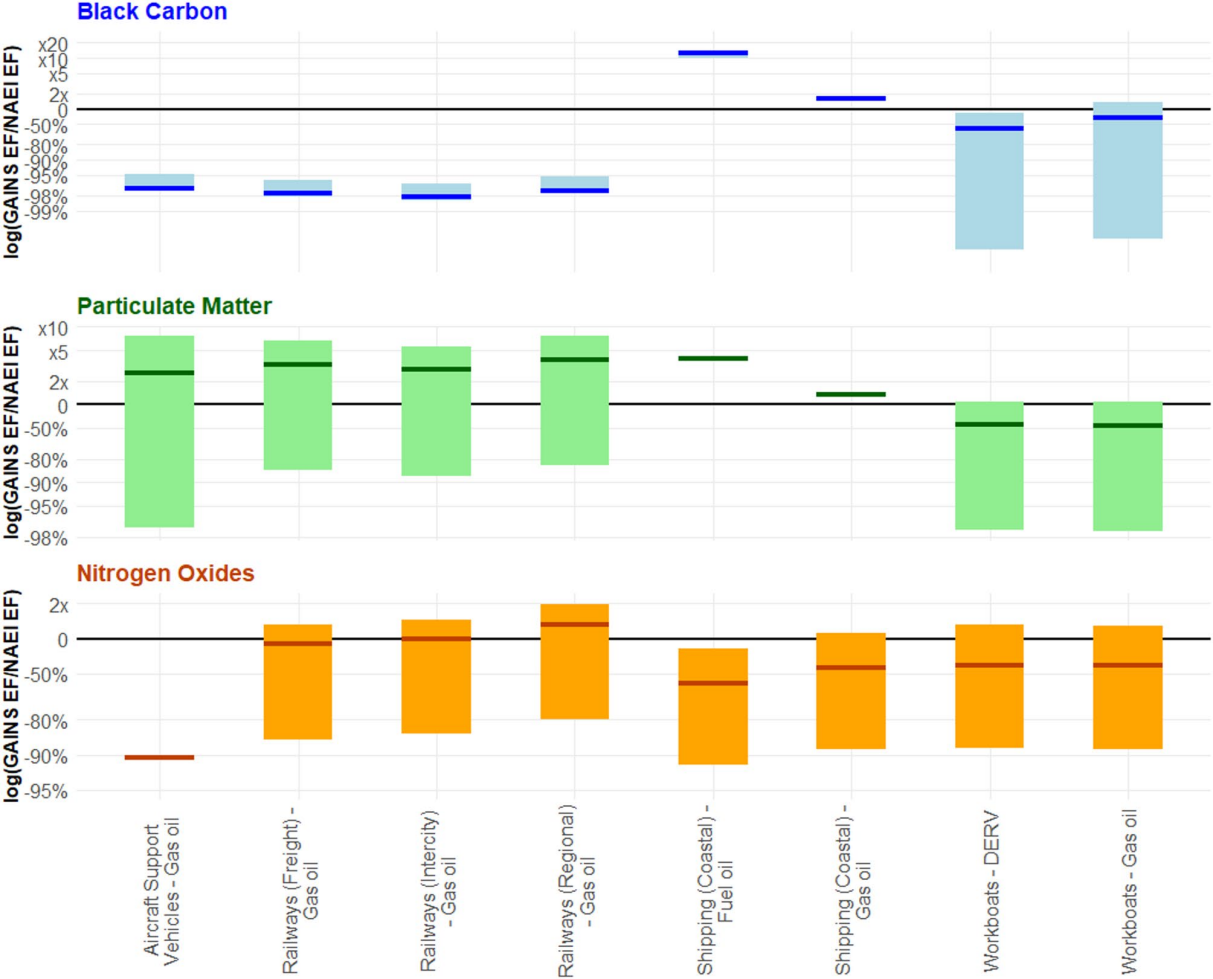
Ratio of GAINS/NAEI emissions factors (EFs), represented on a logarithmic axis, for the remaining combustion sources within 1A3 for black carbon (BC), particulate matter (PM_2.5_), and nitrogen oxides (NO_x_). The y-axis indicates the difference in magnitude between the low, medium, and high GAINS emission factors and the NAEI emission factor. The upper and lower ends of each bar indicate the ratio of the GAINS/NAEI value when applying the highest and lowest GAINS EFs, respectively. The thick coloured line indicates the middle GAINS emission factor compared to the NAEI value. In some cases, the middle and low GAINS emission factors are the same, or only one value is provided– resulting in a single line with no bar. The black line at y = 0 indicates the point at which the GAINS and NAEI emission factors are equal. For the remaining 1A3 combustion sources, the GAINS BC EF range overlaps with the NAEI value on only one source. This is in comparison to 6 of the 8 sources showing overlap between GAINS and NAEI EFs for both PM_2.5_ and NO_x_

**Fig. 8:**
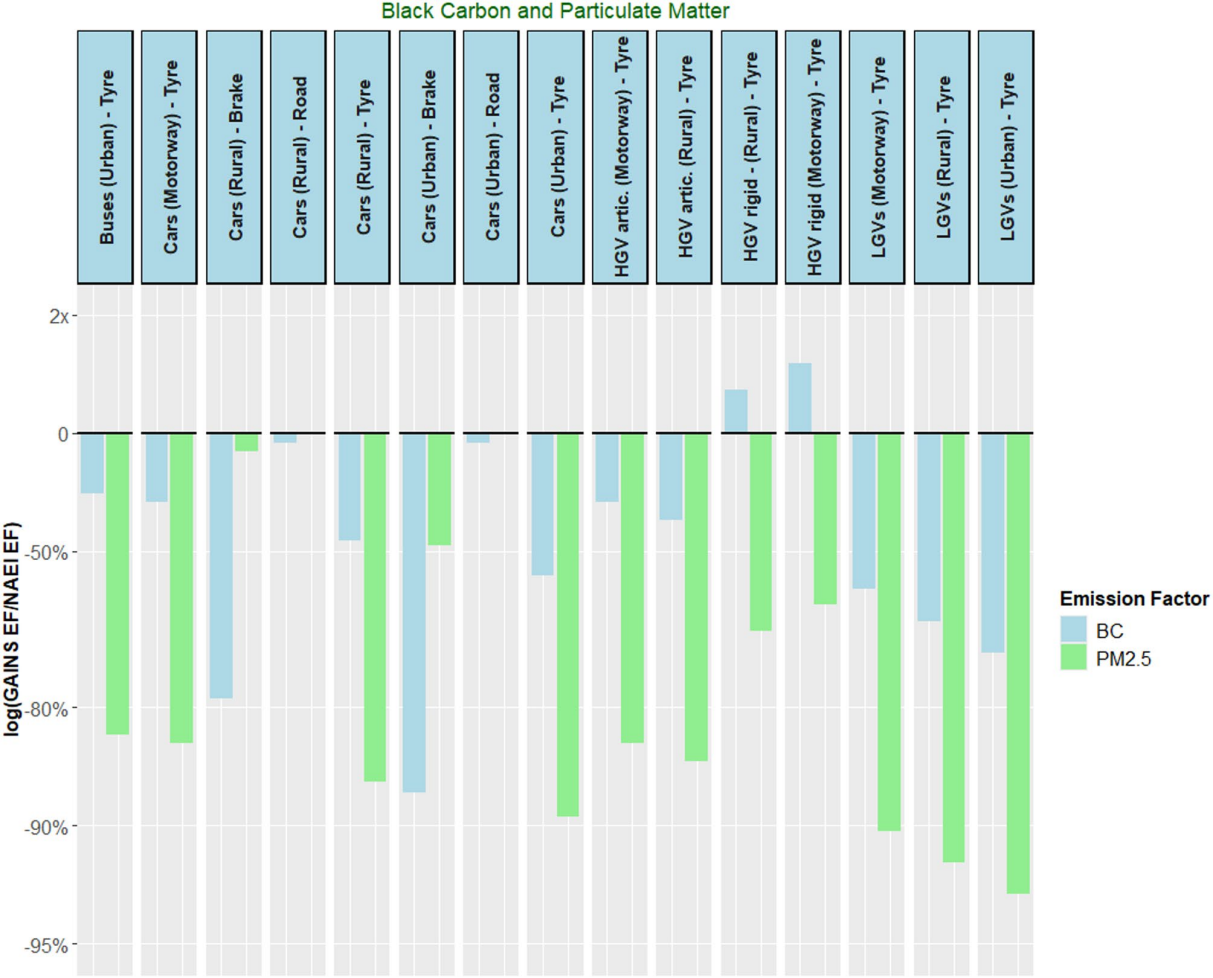
Ratio of GAINS/NAEI emissions factors (EFs), represented on a logarithmic axis, for selected non-exhaust sources within 1A3 for black carbon (BC), particulate matter (PM_2.5_). The y-axis indicates the difference in magnitude between the GAINS and the NAEI emission factors. The solid lines at y = 0 indicate the point at which the GAINS and NAEI emission factors are equal. For the non-exhaust sources within 1A3, GAINS emission factors are consistently smaller when compared to the reference NAEI values across both pollutants, with the BC EFs typically in closer agreement. The two road abrasion sources show exact agreement across PM_2.5_ emission factors and close agreement with BC EFs, also. On some occasions in the GAINS dataset, BC non-exhaust emission factors were found to be larger than those of PM_2.5_. The %BC method used to derive NAEI BC EFs would imply over a 100% share of PM_2.5_ emissions being BC, which is not possible. This is an area that requires further investigation. NB: “- Tyre” denotes an emissions source based on vehicle tyre wear, “- Brake” denotes an emissions source based on vehicle brake wear, and “– Road” denotes an emissions source based on road abrasion

**Fig. 9:**
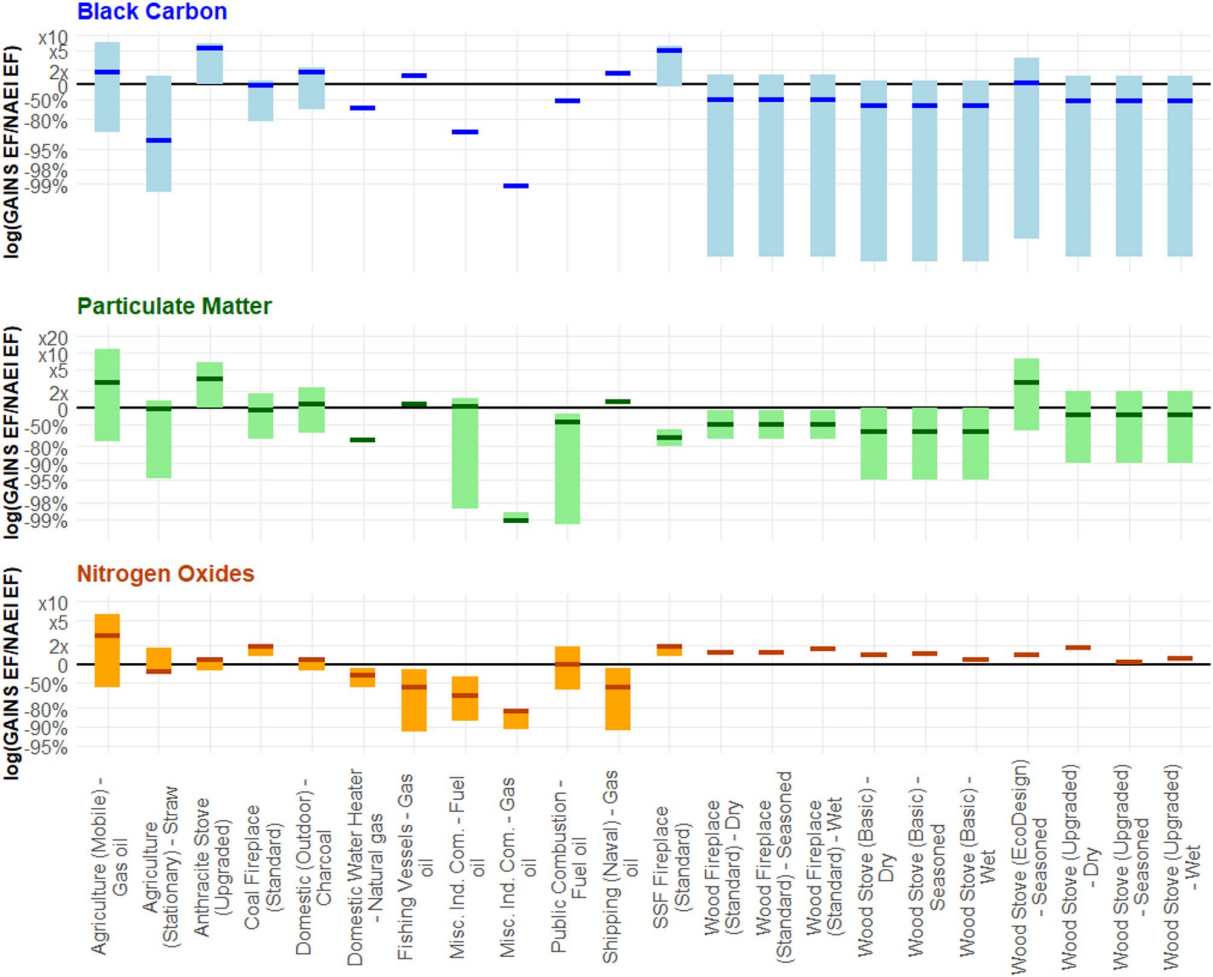
Ratio of GAINS/NAEI emissions factors (EFs), represented on a logarithmic axis, for selected sources within 1A4 and 1A5 for black carbon (BC), particulate matter (PM_2.5_), and nitrogen oxides (NO_x_). The y-axis indicates the difference in magnitude between the low, medium, and high GAINS emission factors and the NAEI emission factor. The upper and lower ends of each bar indicate the ratio of the GAINS/NAEI value when applying the highest and lowest GAINS EFs, respectively. The thick coloured line indicates the middle GAINS emission factor compared to the NAEI value. In some cases, the middle and low GAINS emission factors are the same, or only one value is provided– resulting in a single line with no bar. The black line at y = 0 indicates the point at which the GAINS and NAEI emission factors are equal. For the sources within 1A4 and 1A5, there is overlap and/or close agreement, in the case of domestic wood burning, for all three pollutants

To assess the difference in NAEI and GAINS emission factors, the low, medium, and high GAINS EFs were divided by the corresponding NAEI EF, reflecting the difference in magnitude between the emission factor datasets. For graphical purposes, the logarithmic value was then taken to better represent the discrepancies for both higher and lower values. This provided more space for GAINS values that are much smaller compared to the reference NAEI figure. In the Figs. 4, 5, 6, 7, 8 and 9 and a solid black line is included at y = 0 to indicate where NAEI and GAINS emission factors match exactly.

Some emission factors are excluded from the NAEI database for confidentiality reasons. This was the case for wood combustion in electricity production, for example. Emission factors were derived from wood emissions in the NAEI [14] database and comparing against wood usage within the Digest of UK Energy Statistics [19].

For road transport, the NAEI represents emissions by vehicle type across three different road types: urban, rural, and motorway. These are fleet-aggregated emission factors across each fuel and vehicle type. The GAINS BC EF database is more specific, providing an exhaust EF for each fuel and Euro type. Euro types denote cars that are regulated against different emission standards for cars sold within the UK and Europe. With the introduction of each new Euro type, emissions standards have been increasingly tightened. Euro 1 vehicle standards, introduced in 1992, have much less stringent emissions limits than those for Euro 6 vehicles, first registered in 2015. Instead of assessing the GAINS EFs against the fleet-aggregated NAEI values, they were compared against the Euro-specific EMEP/EEA Guidebook values that the NAEI adopt to produce their fleet-aggregated estimates. In this study, the low EF was taken from Euro 6 vehicles, medium from Euro 4 vehicles, and high from Euro 1 vehicles in both databases. Only one comparison was made for each fuel and vehicle across different Euro types, even when more than one road type (e.g., urban, motorway, or rural) was included for each vehicle and fuel type in the NAEI database. For example, diesel passenger cars contributed more than 0.1% annually to total BC emissions (Sect. Emission factor source selection) across motorway, rural, and urban driving. Whilst these are three distinct sources in the NAEI database, diesel passenger cars were compared only once across the EEA and GAINS databases for Euro 1, Euro 4, and Euro 6 vehicles.

### Emissions comparison method

To contextualise the differences in BC EFs, the three GAINS EF datasets (low, medium, and high) were applied to the same 2021 activity data used by the NAEI to produce their BC emission estimates [14]. The emissions estimates, aggregated by each 1 A NFR sub-sector (1A1 to 1A5), for the GAINS databases were then compared against the NAEI emissions estimates. Road transport emission factors within 1A3 were held constant across GAINS datasets, for the reasons explained in Sect. Emission factor comparison method. The activity data used is included in Table S7 (SI).

## Results and discussion

This section investigates the differences in BC characterisation within the GAINS and NAEI emission factor databases. Parallel comparisons were completed for PM_2.5_ and NO_x_. The investigation is disaggregated according to the Nomenclature for Reporting standardisation. Emission factors are detailed for each source in the supplementary information (Tables S1– S6).

### Energy Industries– 1A1

The 1A1 sector was not a large contributor to the overall BC burden: according to the NAEI [14], the three sources of BC from 1A1 within the largest 83 1 A sources originated from gas oil combustion for upstream oil (1.07%) and gas (0.26%) production, as well as from wood (0.15%) combustion for electricity production.

Looking at gas oil combustion within both (i) upstream oil and (ii) gas production, Fig. 4, the GAINS BC EFs were consistently smaller across the low, medium, and high estimates when compared to the NAEI reference value: for example, the highest GAINS value BC EF was still 97.5% smaller than that of the NAEI. By comparison, the NAEI PM_2.5_ EF for upstream oil production fell between the medium and high PM_2.5_ EFs taken from GAINS. For NO_x_ emissions, the highest GAINS EF remained 86% lower than the NAEI value. For wood combustion in power stations, the NAEI reference values sat in between the low and medium GAINS EFs across all three pollutants, indicating a level of consistency among the EFs for wood combustion. The highest GAINS EFs for both BC and PM_2.5_ were significantly higher than the NAEI value (23x and 58x higher, respectively), which is a result of these being EFs for unabated wood combustion (e.g., with no particulate filters). The NO_x_ comparison was closer between the high GAINS and NAEI estimates (2.4x higher).

### Manufacturing industries and construction– 1A2

1A2 contributed the second most to 1 A BC emissions, the majority of which came from other industrial combustion and non-road mobile machinery, according to the NAEI [14].

Notably, apart from the non-road mobile machinery source, Fig. 5 shows that all industrial GAINS BC EF estimates were smaller than the ones found within the NAEI database, with the differences ranging from 99 to 73% smaller. For “other industrial combustion” of biomass, the largest single source to annual BC emissions (11%), the highest GAINS BC estimate was still 74% lower than the NAEI equivalent.

For both PM_2.5_ and NO_x_, however, the picture is different. Whilst only one set of NAEI BC EFs sat within the GAINS range, 11 and 12 of the 17 PM_2.5_ and NO_x_ NAEI EFs fell between the GAINS values, respectively. Once more, for the important source of “other industrial combustion” of biomass, there was only a 1% and 3% difference between the NAEI and middle GAINS PM_2.5_ and NO_x_ EFs, respectively. This suggests that, for industrial activities, there is a stronger consensus and understanding of PM_2.5_ and NO_x_ EFs, two pollutants with standardised measurement techniques, than there is for BC.

#### Transport (Exhaust Emissions)– 1A3

The 1A3 NFR code had the largest contribution to total 1 A BC emissions, as well as the largest number of sources within the 83 selected. The majority of these came from both exhaust and non-exhaust sources within road transport, with other contributions from shipping, rail, and aviation support vehicles. For the comparison against NO_x_ EFs, only combustion/exhaust-based EFs could be used, and cold starts were excluded from the comparison as these are not included in the GAINS database. For road transport exhaust sources, as explained in Sect. Emission factor comparison method, the NAEI uses EEA Guidebook road transport EFs, which provide values for each Euro vehicle type. This allowed for a direct comparison of Euro-specific road transport EFs for the vehicle and fuel types that contributed most to annual BC emissions.

Looking across the road transport exhaust EF comparison in Fig. 6, the GAINS EFs across all three pollutants were typically smaller than the EEA/NAEI value. Across the 54 different comparisons (18 sources with 3 pollutants each), the GAINS EF was larger than the NAEI value on only 9 occasions. For 7 of the 18 sources, all the BC, PM_2.5_, and NO_x_ EFs were at least 50% smaller than the values provided by the NAEI/EEA. Six of these seven instances came from Euro 1, 4, and 6 articulated and rigid heavy goods vehicles. BC and PM_2.5_ EFs compared very similarly across both databases for 15 of the 18 sources, with discrepancies observed for Euro 4 diesel cars, and Euro 4 and 6 diesel light goods vehicles. The NO_x_ values were in closest agreement across 11 out of the 18 comparisons, but poorer agreement was seen for petrol cars (Euro 1, 4, and 6) and both Euro 6 heavy goods vehicles.

For the other combustion sources in 1A3, aviation support vehicles, rail, and shipping, the NAEI BC EFs only fell within the range of the GAINS BC EFs on one occasion, compared to six for both NO_x_ and PM_2.5_ (Fig. 7). For both rail and aviation support vehicles, the highest GAINS value remained still 95% smaller than the NAEI EF. Closer agreement with these sources was observed across both PM_2.5_ and NO_x_ EFs. A similar pattern was found for the two shipping sources, with the closest GAINS BC EFs being at least 1.6 and 9.8x higher than the NAEI. This was in comparison to 1.3 and 3.7x higher for the PM_2.5_ EFs and 1.1x higher and 18% lower for the NO_x_ EFs.

#### Transport (Non-Exhaust Emissions)– 1A3

The non-exhaust emissions from road transport were also sources of BC and PM_2.5_. In the NAEI and GAINS databases, only one BC and PM_2.5_ EF was available per source. Here, a discrepancy was observed between the databases’ PM2.5 and BC EFs from road transport non-exhaust sources, as shown in Fig. 8. This discrepancy was most pronounced when examining brake wear and tyre wear sources, where the NAEI sources consistently exceeded those from GAINS, except for rigid HGV tyre wear BC emissions. The road abrasion sources were in close agreement, with a discrepancy of less than 6% between BC EFs and a perfect match with PM_2.5_ EFs. Across the non-exhaust sources investigated, BC values were consistently closer across the two datasets than for PM_2.5_. The average difference among the non-exhaust BC EFs was around 36%, whereas the PM_2.5_ EFs were 65% smaller than the NAEI value.

A potential reason for the discrepancy in non-exhaust EFs is that there are several occasions in the GAINS EF dataset where BC EFs were larger than those of the PM_2.5_ EFs. This implies that for the %BC method to align, BC would have to be more than 100% of PM_2.5_ content. For example, PM_2.5_ tyre wear emissions from rigid HGVs in the GAINS database were set at 0.0042 kt Gvkm^− 1^, whereas the BC value was 0.0063 kt Gvkm^− 1^– 50% larger than the PM_2.5_ value. This is an area of contention that requires more investigation in the future.

### Other sectors– 1A4 and 1A5

BC sources from 1A4 and 1A5 were found in domestic, public and commercial combustion, agricultural machinery, and naval shipping. The largest sources originated from domestic wood combustion and the use of gas oil in agricultural mobile machinery.

Looking at the two EF databases in Fig. 9, there was more similarity between the EFs in 1A4 and 1A5 sources in comparison to other 1 A sectors. For domestic wood combustion sources, the EFs aligned well across different pollutants. Across both BC and PM_2.5_, the NAEI reference value fell between the middle and high GAINS EF, except for the ‘EcoDesign’ stove, which sat slightly below the medium in both cases. Only one NO_x_ EF was provided for wood combustion, yet the GAINS value in remained less than 1.8x higher across all instances. Within agricultural mobile machinery, there was no discernible difference in the agreement of NAEI and GAINS EFs across all three pollutants.

Public sector and miscellaneous combustion sources showed GAINS BC EFs to be smaller than NAEI estimates. Across all three sources, the NAEI and GAINS values were in closer agreement for both PM_2.5_ and NO_x_ than for BC.

### Emissions comparison

**Fig. 10:**
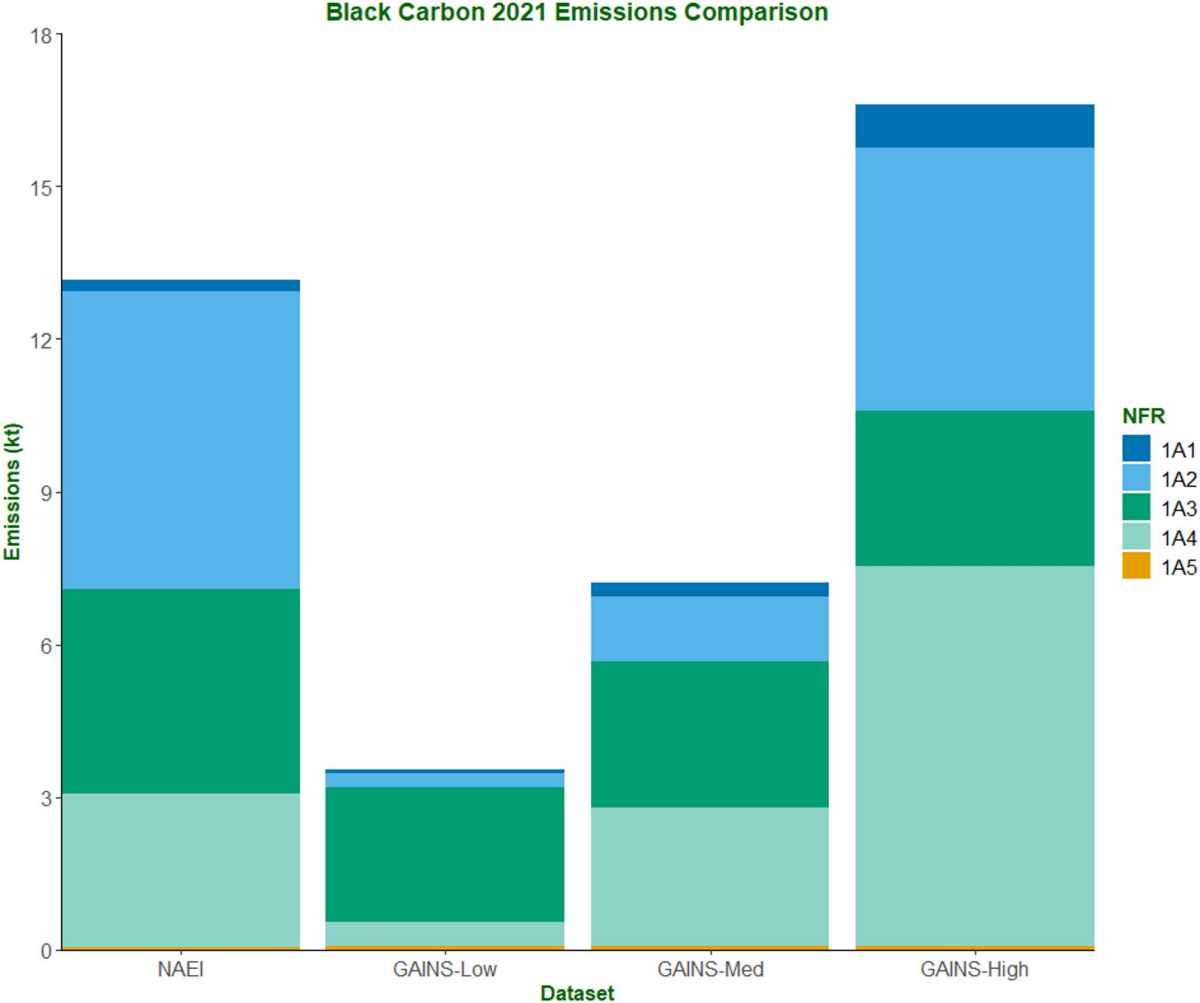
2021 BC emissions estimates (kt) across 1 A sources (broken down by NFR sub-sectors) for the NAEI and three GAINS EF datasets

Figure 10 presents the 2021 BC emissions estimates for the NAEI and three GAINS datasets, using 2021 NAEI activity data. Overall, BC emissions in 2021 within 1 A totalled 13.2 kt, whilst the GAINS estimates ranged from 3.5 to 16.6 kt. Two main sectors were responsible for driving the differences in emissions: 1A2 (manufacturing industries and construction) and 1A4 (commercial, residential, and agricultural combustion). Within 1A2, NAEI BC emissions were reported as 5.8 kt in 2021. The lowest and middle GAINS datasets estimated 0.3 and 1.3 kt, respectively, whilst the highest GAINS dataset estimated 5.2 kt. The largest source of difference between the NAEI and the highest GAINS dataset within 1A2 resulted from ‘other industrial combustion’ of biomass (2.7 kt– NAEI; 0.7 kt– GAINS). Across 1A2, NAEI emissions are consistently higher than the highest GAINS BC estimates. However, the difference in 1A2 BC emissions is masked by the highest GAINS emission factor for industrial off-road mobile machinery, estimating around 3.2 kt compared to the NAEI figure of 0.5 kt.

Looking at 1A4, the middle GAINS BC EFs (2.7 kt) yielded similar BC emissions to the NAEI (3.0 kt). The highest GAINS estimate of BC emissions within 1A4 (7.5 kt) was primarily due to higher estimates of BC emissions from gas oil-based agricultural mobile machinery (0.4 kt NAEI; 2.8 kt GAINS) and domestic wood combustion (1.9 kt NAEI; 3.2 kt GAINS).

The road transport sources within 1A3 contributed 78% of the BC emissions within the NAEI estimates (3.1 kt) and between 62% and 72% (1.9 kt) in the GAINS estimates, depending on the EFs used. 1A1 contributed minimally (0.2 kt NAEI; 0.1–0.9 kt GAINS) and 1A5 contributed less than 0.1 kt across all databases.

### Discussion

Section Energy Industries– 1A1–Other sectors– 1A4 and 1A5 illustrate the similarities and differences between GAINS and NAEI BC, PM_2.5_, and NO_x_ emission factors. Across the sectors where a single (technology-aggregated) NAEI value was compared against low, medium, and high GAINS emission factors, 32 out of the 50 sources (64%) saw the GAINS BC emission factor range sit outside the NAEI reference. By comparison, this value was 26% for PM_2.5_ and 46% for NO_x_. For road transport emission factors, non-exhaust emission factors and Euro-specific emission factors were compared on a like-for-like basis. Across the 18 exhaust-based road transport emission factors, the GAINS EFs were lower in 45 of the 54 (18 sources, comparing 3 pollutants per source) comparisons. BC and PM_2.5_ emission factors compared closely in 14 of the 18 sources. It appears that, in a sector as strongly regulated as road transport, there is more consistency between the three pollutants in each database. Yet, comparisons between databases are still poor. For the 15 non-exhaust road transport sources, the BC EFs showed closer agreement than PM_2.5_ in 11 of them. In some cases, such as tyre wear emissions from heavy goods vehicles, the GAINS BC emission factor was seen to be larger than the corresponding PM_2.5_ value. In the NAEI database, this would not be possible using the %BC method, as this would imply a value of more than 100% of the PM_2.5_ fraction is the BC component. This is an area that warrants further investigation.

The largest area of disagreement was between BC emission factors within 1A2 (Manufacturing Industries and Construction) and, importantly, in ‘other industrial combustion’ of biomass: GAINS BC EFs were found to be between 73% and 99% lower than the NAEI estimates. Contrastingly, the PM_2.5_ and NO_x_ EFs for the same source showed much more consistency, with the middle EFs differing by only 1% and 2%, respectively. This is an important finding considering that biomass use in ‘other industrial combustion’ contributed over 11% of total BC emissions from 2015 to 2021, according to the NAEI– the largest single source in the inventory.

Section Emissions comparison investigated 2021 BC emissions estimates for the four datasets to see how the different emission factors compared with the same 2021 NAEI activity data. The largest areas of BC emissions variance were found within NFR codes 1A2 (manufacturing industries and construction) and 1A4 (commercial, residential, and agricultural combustion). As a result, both 1A2 and 1A4 should be the areas that policymakers and researchers should focus on in the future to best constrain these emissions. The other sub-sectors within 1 A appear to be better constrained or contribute minimally to overall BC emissions.

There is a clear lack of consensus between the BC emission factors within the NAEI and GAINS databases, more so than either PM_2.5_ or NO_x_. This may be expected, given that BC emissions are not measured using a standardised and regulated method. As a result, it is not possible to infer which of the two databases, created using different techniques, portrays the most accurate emission factors. The reliance on PM_2.5_ EFs in some sectors using the NAEI method may notably impact BC EFs. However, as shown above, for the key source of ‘other industrial combustion’ of biomass, PM_2.5_ EFs appear to align well, and discrepancies in BC EFs remain. This suggests that the %BC fraction can still be a key contributor to uncertainty in these EFs.

The absence of clarity presents many challenges for countries wanting to report BC emissions potentially under the Gothenburg Protocol, especially in the wake of experts calling for more holistic thinking between climate and air pollution communities for climate-forcing pollutants like BC [11]. Air pollution abatement and international emissions negotiations require reliable inventories and source apportionment. In the case of BC, it is also important to understand how measures to reduce PM_2.5_ emissions will address BC emissions. This may not be possible with the current inventories and measurement techniques available.

## Conclusions

The UK’s black carbon (BC) emissions are uncertain, with the National Atmospheric Emissions Inventory (NAEI) assigning a 20% and 50% uncertainty range to annual emissions. Accurate measurement of a problem is the first step towards mitigating it, which is critically important given the negative impacts of BC on both human health and the environment. One reason for this considerable uncertainty is the lack of a standardised measurement technique for assessing BC emissions. This paper reviews the UK emission factors used by the NAEI to estimate BC emissions, which use a fraction of PM_2.5_ technique, against mass-based BC emission factors from the Greenhouse Gas Interactions and Synergies (GAINS) model. PM_2.5_ and NO_x_ emission factors from these two datasets were also compared in a similar manner. These are two pollutants that have standardised measurement techniques and, in the case of PM_2.5_ emissions, contribute to NAEI BC estimates.

The comparison of emission factors was limited to Nomenclature for Reporting (NFR) code 1 A (Energy Industries) as it contributed 77% of all UK BC emissions from 2015 to 2021, according to the NAEI [14]. BC emission factors across the two databases did not compare well with one another. When the technology-aggregated NAEI emission factors were compared against GAINS technologies with specific abatement measures, the NAEI value fell outside of the range of the low, medium, and high GAINS emission factors 64% of the time. By contrast, for PM_2.5_ and NO_x_, this value dropped to 26% and 46%, respectively. When reviewing road transport emission factors, BC compares similarly to both PM_2.5_ and NO_x_. The poorest BC comparisons were found within manufacturing industries and construction sources, where the highest GAINS BC emission factor for “other industrial combustion” of biomass remained 73% lower than the corresponding NAEI value. This was the largest source in the NAEI database, accounting for approximately 11% of the total UK BC emissions annually. The 2021 NAEI activity data was applied to these four emission factors sets to produce BC emissions estimates for 2021. The comparison found the largest sources of variability within 1A2 (manufacturing industries and construction) and 1A4 (commercial, residential, and agricultural combustion). This suggests that future research should focus on constraining these emission sources. The remaining sub-sectors within 1 A were better constrained or did not contribute largely to BC emissions.

Moving to a mass-based BC emission factor database may provide benefits for reducing BC EF uncertainty, as it removes the dependency on both PM_2.5_ emission factors and the factor describing the BC content within PM_2.5_. This is not certain, however, given the lack of a standardised measurement technique for assessing BC emissions, which makes it challenging to determine the ‘true’ value of a BC emission factor. Other potential actions to reduce BC emission uncertainty and improve comparison could include improving PM_2.5_ emission factors in sectors that currently compare poorly, given that PM_2.5_ emissions use standardised measurement techniques and are subject to regulatory measurement programmes. More research is needed to align these BC emission factors, allowing us to reduce the environmental and health burdens of BC in a more effective and targeted manner.

## Electronic supplementary material

Below is the link to the electronic supplementary material.

Supplementary Material 1: The following supplementary information can be found at:. Table S1: Black carbon emission factors from the NAEI and GAINS datasets. Table S2: Non-exhaust black carbon and PM_2.5_ road transport emission factors from the GAINS and NAEI (EMEP/EEA) databases. Table S3: Exhaust-based black carbon, PM_2.5_, and NO_x_ road transport emission factors from the GAINS and NAEI (EMEP/EEA) databases. Table S4: NAEI exhaust-based road transport contributions to total annual BC emissions, by source. Table S5: PM_2.5_ emission factors from the NAEI and GAINS datasets. Table S6: NO_x_ emission factors from the NAEI and GAINS datasets. Table S7: 2021 NAEI activity data for BC emissions estimates. Figure S1: Number of sources within each NAEI 1 A NFR code (sub-sector) with an annual average contribution to BC emissions above 0.1%.

## Data Availability

No datasets were generated or analysed during the current study.
