# Supplementary material for: Large differences between UK black carbon emission factors
Source: Carbon Balance Manag. 2025 Jul 2;20:19. doi: 10.1186/s13021-025-00306-3 (PMC12224827; doi:10.1186/s13021-025-00306-3)

**Black Carbon Supplementary Information**

**Black Carbon, PM_2.5_, and NO_x_ Emission Factors**

Table S1: Black carbon (BC) emission factors for 1A (energy industries) sources from the GAINS (low, medium, and high estimates) and NAEI emission factor databases. Also included is the average % contribution to total (including other sectors aside from 1A) BC emissions from 2015 - 2021, according to the NAEI. Only sources with a contribution above 0.1% are included.

|  | | | | **BC EFs (kg/TWh)** | | | |
| --- | --- | --- | --- | --- | --- | --- | --- |
| **NFR Code** | **Source** | **Activity** | **Average contribution to total BC (%)** | **NAEI** | **GAINS Low** | **GAINS Medium** | **GAINS High** |
| 1A1a | Power stations | Wood | 0.15 | 391 | 360 | 792 | 8892 |
| 1A1c | Upstream Oil Production - fuel combustion | Gas oil | 1.07 | 26144 | 108 | 612 | 648 |
| 1A1c | Upstream Gas Production - fuel combustion | Gas oil | 0.26 | 71938 | 108 | 612 | 648 |
| 1A2a | Iron and steel - combustion plant | Fuel oil | 0.61 | 381655 | 3996 | 3996 | 4212 |
| 1A2c | Chemicals (combustion) | Biomass | 0.17 | 135518 | 2124 | 3168 | 35532 |
| 1A2c | Chemicals (combustion) | Fuel oil | 0.15 | 80640 | 6516 | 6516 | 6876 |
| 1A2d | Pulp, Paper and Print (combustion) | Biomass | 3.42 | 135518 | 2124 | 3168 | 35532 |
| 1A2d | Pulp, Paper and Print (combustion) | Coal | 0.24 | 40409 | 360 | 504 | 5760 |
| 1A2e | Food & drink, tobacco (combustion) | Biomass | 0.50 | 135518 | 2124 | 3168 | 35532 |
| 1A2e | Food & drink, tobacco (combustion) | Coal | 0.16 | 40386 | 360 | 540 | 6012 |
| 1A2f | Mineral products (other): combustion | Biomass | 1.89 | 135518 | 2124 | 3168 | 35532 |
| 1A2gvii | Industrial off-road mobile machinery | Gas oil | 5.19 | 30007 | 4536 | 45324 | 177408 |
| 1A2gviii | Other industrial combustion | Biomass | 11.25 | 135518 | 2124 | 3168 | 35532 |
| 1A2gviii | Other industrial combustion | Burning oil | 5.22 | 40320 | 3996 | 3996 | 4212 |
| 1A2gviii | Other industrial combustion | Wood | 3.41 | 141120 | 2124 | 3168 | 35532 |
| 1A2gviii | Other industrial combustion | Coal | 1.56 | 40409 | 36 | 72 | 648 |
| 1A2gviii | Other industrial combustion | Fuel oil | 0.82 | 69588 | 3996 | 3996 | 4212 |
| 1A2gviii | Other industrial combustion | Lubricants | 0.41 | 51010 | 360 | 360 | 396 |
| 1A2gviii | Other industrial combustion | Gas oil | 0.26 | 51010 | 360 | 360 | 396 |
| 1A2gviii | Other industrial combustion | Petroleum coke | 0.12 | 40409 | 360 | 360 | 396 |
| 1A3c | Railways - intercity | Gas oil | 0.66 | 40460 | 661 | 744 | 1408 |
| 1A3c | Railways - regional | Gas oil | 0.60 | 29840 | 661 | 744 | 1408 |
| 1A3c | Railways - freight | Gas oil | 0.39 | 34368 | 661 | 744 | 1408 |
| 1A3dii | Shipping - coastal | Gas oil | 2.54 | 23083 | 37080 | 37080 | 38088 |
| 1A3dii | Motorboats / workboats | DERV | 1.96 | 189546 | 324 | 77688 | 155412 |
| 1A3dii | Motorboats / workboats | Gas oil | 0.37 | 115212 | 324 | 77688 | 155412 |
| 1A3dii | Shipping - coastal | Fuel oil | 0.33 | 13748 | 135000 | 170748 | 175500 |
| 1A3eii | Aircraft - support vehicles | Gas oil | 0.52 | 26380 | 661 | 744 | 1408 |
| 1A4ai | Misc. industrial/commercial combustion | Fuel oil | 0.16 | 36288 | 3996 | 3996 | 4212 |
| 1A4ai | Public sector combustion | Fuel oil | 0.14 | 69120 | 32616 | 32616 | 34344 |
| 1A4ai | Misc. industrial/commercial combustion | Gas oil | 0.11 | 39127 | 360 | 360 | 396 |
| 1A4bi | Domestic Closed Stove - Upgraded | Wood - Seasoned | 3.70 | 213120 | 72 | 100800 | 328752 |
| 1A4bi | Domestic Fireplace - Standard | Coal | 1.85 | 116887 | 21204 | 113040 | 141264 |
| 1A4bi | Domestic Closed Stove - Upgraded | Wood - Wet | 1.73 | 213120 | 72 | 100800 | 328752 |
| 1A4bi | Domestic Fireplace - Standard | Wood - Seasoned | 1.68 | 206640 | 72 | 100800 | 328752 |
| 1A4bi | Domestic Closed Stove - Upgraded | Wood - Dry | 1.20 | 213120 | 72 | 100800 | 328752 |
| 1A4bi | Domestic Fireplace - Standard | Wood - Wet | 0.78 | 206640 | 72 | 100800 | 328752 |
| 1A4bi | Domestic Closed Stove - Basic | Wood - Seasoned | 0.74 | 266400 | 72 | 100800 | 328752 |
| 1A4bi | Domestic Fireplace - Standard | Wood - Dry | 0.54 | 206640 | 72 | 100800 | 328752 |
| 1A4bi | Domestic Outdoor | Charcoal | 0.40 | 64780 | 21204 | 113040 | 141264 |
| 1A4bi | Domestic Closed Stove - Basic | Wood - Wet | 0.35 | 266400 | 72 | 100800 | 328752 |
| 1A4bi | Domestic Closed Stove - Basic | Wood - Dry | 0.24 | 266400 | 72 | 100800 | 328752 |
| 1A4bi | Domestic Closed Stove - EcoDesign | Wood - Seasoned | 0.21 | 93744 | 72 | 100800 | 328752 |
| 1A4bi | Domestic Water Heater | Natural gas | 0.19 | 428 | 144 | 144 | 144 |
| 1A4bi | Domestic Fireplace - Standard | SSF | 0.13 | 23377 | 21204 | 113040 | 141264 |
| 1A4bi | Domestic Closed Stove - Upgraded | Anthracite | 0.12 | 20736 | 21204 | 113040 | 141264 |
| 1A4ci | Agriculture - stationary combustion | Straw | 0.80 | 141491 | 972 | 10800 | 216000 |
| 1A4cii | Agriculture - mobile machinery | Gas oil | 4.63 | 22178 | 2520 | 40176 | 157176 |
| 1A4ciii | Fishing vessels | Gas oil | 0.37 | 24429 | 37080 | 37080 | 38088 |
| 1A5b | Shipping - naval | Gas oil | 0.30 | 21791 | 37080 | 37080 | 38088 |

Table S2: Non-exhaust black carbon (BC) and PM_2.5_ emission factors for road transport sources from the GAINS and NAEI emission factor databases. Only one value is provided in each database, so no low, medium, and high GAINS estimates are included. Also included is the average % contribution to total (including other sectors aside from 1A) BC emissions from 2015 - 2021, according to the NAEI. Only sources with a contribution above 0.1% are included.

| **NFR/CRF Group** | **Source** | **Activity** | **Average contribution to total BC (%)** | **NAEI BC EF (g/km)** | **GAINS BC EF (g/km)** | **NAEI PM25 EF (g/km)** | **GAINS PM25 (g/km)** |
| --- | --- | --- | --- | --- | --- | --- | --- |
| 1A3bvi | Road transport - cars - rural driving | Tyre wear | 2.41 | 0.0019 | 0.0010 | 0.0051 | 0.0007 |
| 1A3bvi | Road transport - cars - urban driving | Tyre wear | 2.39 | 0.0023 | 0.0010 | 0.0062 | 0.0007 |
| 1A3bvi | Road transport - LGVs - rural driving | Tyre wear | 0.85 | 0.0030 | 0.0010 | 0.0082 | 0.0007 |
| 1A3bvi | Road transport - cars - motorway driving | Tyre wear | 0.80 | 0.0015 | 0.0010 | 0.0041 | 0.0007 |
| 1A3bvi | Road transport - LGVs - urban driving | Tyre wear | 0.68 | 0.0036 | 0.0010 | 0.0099 | 0.0007 |
| 1A3bvi | Road transport - HGV articulated - motorway driving | Tyre wear | 0.59 | 0.0094 | 0.0063 | 0.0259 | 0.0042 |
| 1A3bvi | Road transport - HGV articulated - rural driving | Tyre wear | 0.42 | 0.0105 | 0.0063 | 0.0288 | 0.0042 |
| 1A3bvi | Road transport - cars - urban driving | Brake wear | 0.34 | 0.0003 | 0.0000 | 0.0049 | 0.0026 |
| 1A3bvi | Road transport - LGVs - motorway driving | Tyre wear | 0.29 | 0.0025 | 0.0010 | 0.0068 | 0.0007 |
| 1A3bvi | Road transport - cars - rural driving | Brake wear | 0.25 | 0.0002 | 0.0000 | 0.0028 | 0.0026 |
| 1A3bvi | Road transport - HGV rigid - rural driving | Tyre wear | 0.21 | 0.0049 | 0.0063 | 0.0134 | 0.0042 |
| 1A3bvii | Road transport - cars - rural driving | Road abrasion | 0.21 | 0.0002 | 0.0002 | 0.0041 | 0.0041 |
| 1A3bvii | Road transport - cars - urban driving | Road abrasion | 0.17 | 0.0002 | 0.0002 | 0.0041 | 0.0041 |
| 1A3bvi | Road transport - buses and coaches - urban driving | Tyre wear | 0.14 | 0.0090 | 0.0063 | 0.0246 | 0.0042 |
| 1A3bvi | Road transport - HGV rigid - motorway driving | Tyre wear | 0.12 | 0.0042 | 0.0063 | 0.0115 | 0.0042 |

Table S3: Exhaust-based black carbon (BC), PM_2.5_, and NO_x_ emission factors for road transport sources from the GAINS and NAEI emission factor databases. Euro-specific GAINS emission factors were compared against Euro-specific EMEP/EEA Guidebook values, which are used by the NAEI to produce their fleet-aggregated estimates. This allows for a better comparison than when using the NAEI aggregated emission factors.

| **NFR Group** | **Source** | **Activity** | **NAEI BC EF (g/km)** | **GAINS BC EF (g/km)** | **NAEI PM_2.5_ EF (g/km)** | **GAINS PM_2.5_ (g/km)** | **NAEI NO_x_ EF (g/km)** | **GAINS NO_x_ (g/km)** |
| --- | --- | --- | --- | --- | --- | --- | --- | --- |
| 1A3biii | Road transport - buses and coaches - Euro 1 | DERV | 0.3114 | 0.0999 | 0.4790 | 0.1552 | 7.39 | 4.18 |
| 1A3biii | Road transport - buses and coaches - Euro 4 | DERV | 0.0347 | 0.0101 | 0.0462 | 0.0135 | 4.18 | 2.73 |
| 1A3biii | Road transport - buses and coaches - Euro 6 | DERV | 0.0003 | 0.0001 | 0.0023 | 0.0007 | 0.20 | 0.47 |
| 1A3bi | Road transport - cars - Euro 1 | DERV | 0.0589 | 0.0166 | 0.0842 | 0.0237 | 0.56 | 0.24 |
| 1A3bi | Road transport - cars - Euro 4 | DERV | 0.0031 | 0.0109 | 0.0314 | 0.0126 | 0.47 | 0.31 |
| 1A3bi | Road transport - cars - Euro 6 | DERV | 0.0002 | 0.0001 | 0.0015 | 0.0007 | 0.04 | 0.08 |
| 1A3bi | Road transport - cars - Euro 1 | Petrol | 0.0006 | 0.0007 | 0.0022 | 0.0029 | 0.26 | 0.14 |
| 1A3bi | Road transport - cars - Euro 4 | Petrol | 0.0002 | 0.0001 | 0.0011 | 0.0008 | 0.05 | 0.01 |
| 1A3bi | Road transport - cars - Euro 6 | Petrol | 0.0002 | 0.0002 | 0.0016 | 0.0013 | 0.02 | 0.01 |
| 1A3biii | Road transport - HGV articulated - Euro 1 | DERV | 0.1931 | 0.0698 | 0.2970 | 0.1084 | 7.60 | 2.96 |
| 1A3biii | Road transport - HGV articulated - Euro 4 | DERV | 0.0179 | 0.0071 | 0.0239 | 0.0094 | 4.47 | 1.77 |
| 1A3biii | Road transport - HGV articulated - Euro 6 | DERV | 0.0002 | 0.0001 | 0.0012 | 0.0005 | 0.24 | 0.06 |
| 1A3biii | Road transport - HGV rigid - Euro 1 | DERV | 0.1931 | 0.0698 | 0.2970 | 0.1084 | 7.40 | 2.96 |
| 1A3biii | Road transport - HGV rigid - Euro 4 | DERV | 0.0179 | 0.0071 | 0.0239 | 0.0094 | 4.46 | 1.77 |
| 1A3biii | Road transport - HGV rigid - Euro 6 | DERV | 0.0002 | 0.0001 | 0.0012 | 0.0005 | 0.28 | 0.06 |
| 1A3bii | Road transport - LGVs - Euro 1 | DERV | 0.0819 | 0.0241 | 0.1170 | 0.0345 | 1.04 | 0.55 |
| 1A3bii | Road transport - LGVs - Euro 4 | DERV | 0.0041 | 0.0159 | 0.0409 | 0.0183 | 0.70 | 0.44 |
| 1A3bii | Road transport - LGVs - Euro 6 | DERV | 0.0001 | 0.0002 | 0.0009 | 0.0010 | 0.08 | 0.12 |

Table S4: Average annual (2015 – 2021) contribution to UK BC emissions from exhaust-based road transport sources, according to the NAEI. These are fleet-aggregated sources, which were expanded upon using Euro-specific emission factors in Table S3 and used effectively as a scoping exercise.

| **NFR/CRF Group** | **Source** | **Activity** | **Average contribution to total BC (%)** |
| --- | --- | --- | --- |
| 1A3bi | Road transport - cars - rural driving | DERV | 3.50 |
| 1A3bi | Road transport - cars - urban driving | DERV | 3.29 |
| 1A3bi | Road transport - cars - cold start | DERV | 3.11 |
| 1A3bii | Road transport - LGVs - rural driving | DERV | 1.91 |
| 1A3bi | Road transport - cars - motorway driving | DERV | 1.83 |
| 1A3bii | Road transport - LGVs - urban driving | DERV | 1.70 |
| 1A3bii | Road transport - LGVs - motorway driving | DERV | 1.18 |
| 1A3bii | Road transport - LGVs - cold start | DERV | 1.01 |
| 1A3biii | Road transport - HGV rigid - rural driving | DERV | 0.87 |
| 1A3biii | Road transport - HGV articulated - motorway driving | DERV | 0.71 |
| 1A3biii | Road transport - buses and coaches - urban driving | DERV | 0.57 |
| 1A3biii | Road transport - HGV rigid - motorway driving | DERV | 0.50 |
| 1A3biii | Road transport - HGV articulated - rural driving | DERV | 0.50 |
| 1A3biii | Road transport - HGV rigid - urban driving | DERV | 0.49 |
| 1A3biii | Road transport - buses and coaches - rural driving | DERV | 0.20 |
| 1A3bi | Road transport - cars - urban driving | Petrol | 0.13 |
| 1A3biii | Road transport - HGV articulated - urban driving | DERV | 0.12 |
| 1A3bi | Road transport - cars - rural driving | Petrol | 0.10 |

Table S5: PM_2.5_ emission factors for 1A (energy industries) sources from the GAINS (low, medium, and high estimates) and NAEI emission factor databases that have greater than a 0.1% contribution to black carbon (BC) emissions (see table S1 caption).

|  | | | | **PM2.5 EFs (kt/TWh)** | | | |
| --- | --- | --- | --- | --- | --- | --- | --- |
| **NFR Code** | **Source** | **Activity** | **Average contribution to total BC (%)** | **NAEI** | **GAINS Low** | **GAINS Medium** | **GAINS High** |
| 1A1a | Power stations | Wood | 0.15 | 0.01 | 0.00 | 0.48 | 0.68 |
| 1A1c | Upstream Oil Production - fuel combustion | Gas oil | 1.07 | 0.08 | 0.00 | 0.07 | 0.10 |
| 1A1c | Upstream Gas Production - fuel combustion | Gas oil | 0.26 | 0.21 | 0.00 | 0.07 | 0.10 |
| 1A2a | Iron and steel - combustion plant | Fuel oil | 0.61 | 0.68 | 0.00 | 0.07 | 0.10 |
| 1A2c | Chemicals (combustion) | Biomass | 0.17 | 0.48 | 0.03 | 0.48 | 0.68 |
| 1A2c | Chemicals (combustion) | Fuel oil | 0.15 | 0.14 | 0.00 | 0.07 | 0.10 |
| 1A2d | Pulp, Paper and Print (combustion) | Biomass | 3.42 | 0.48 | 0.01 | 0.48 | 0.68 |
| 1A2d | Pulp, Paper and Print (combustion) | Coal | 0.24 | 0.63 | 0.01 | 0.08 | 1.10 |
| 1A2e | Food & drink, tobacco (combustion) | Biomass | 0.50 | 0.48 | 0.03 | 0.48 | 0.68 |
| 1A2e | Food & drink, tobacco (combustion) | Coal | 0.16 | 0.63 | 0.05 | 0.08 | 1.15 |
| 1A2f | Mineral products (other): combustion | Biomass | 1.89 | 0.48 | 0.03 | 0.48 | 0.68 |
| 1A2gvii | Industrial off-road mobile machinery | Gas oil | 5.19 | 0.05 | 0.01 | 0.09 | 0.36 |
| 1A2gviii | Other industrial combustion | Biomass | 11.25 | 0.48 | 0.03 | 0.48 | 0.68 |
| 1A2gviii | Other industrial combustion | Burning oil | 5.22 | 0.07 | 0.00 | 0.07 | 0.10 |
| 1A2gviii | Other industrial combustion | Wood | 3.41 | 0.50 | 0.03 | 0.48 | 0.68 |
| 1A2gviii | Other industrial combustion | Coal | 1.56 | 0.63 | 0.03 | 0.21 | 3.05 |
| 1A2gviii | Other industrial combustion | Fuel oil | 0.82 | 0.12 | 0.00 | 0.07 | 0.10 |
| 1A2gviii | Other industrial combustion | Lubricants | 0.41 | 0.09 | 0.00 | 0.00 | 0.00 |
| 1A2gviii | Other industrial combustion | Gas oil | 0.26 | 0.09 | 0.00 | 0.00 | 0.00 |
| 1A2gviii | Other industrial combustion | Petroleum coke | 0.12 | 0.63 | 0.00 | 0.34 | 0.48 |
| 1A3c | Railways - intercity | Gas oil | 0.66 | 0.06 | 0.01 | 0.17 | 0.35 |
| 1A3c | Railways - regional | Gas oil | 0.60 | 0.05 | 0.01 | 0.17 | 0.35 |
| 1A3c | Railways - freight | Gas oil | 0.39 | 0.05 | 0.01 | 0.17 | 0.35 |
| 1A3dii | Shipping - coastal | Gas oil | 2.54 | 0.07 | 0.09 | 0.09 | 0.09 |
| 1A3dii | Motorboats / workboats | DERV | 1.96 | 0.34 | 0.01 | 0.19 | 0.38 |
| 1A3dii | Motorboats / workboats | Gas oil | 0.37 | 0.35 | 0.01 | 0.19 | 0.38 |
| 1A3dii | Shipping - coastal | Fuel oil | 0.33 | 0.10 | 0.41 | 0.41 | 0.41 |
| 1A3eii | Aircraft - support vehicles | Gas oil | 0.52 | 0.04 | 0.00 | 0.11 | 0.32 |
| 1A4ai | Misc. industrial/commercial combustion | Fuel oil | 0.16 | 0.06 | 0.00 | 0.07 | 0.10 |
| 1A4ai | Public sector combustion | Fuel oil | 0.14 | 0.12 | 0.00 | 0.07 | 0.10 |
| 1A4ai | Misc. industrial/commercial combustion | Gas oil | 0.11 | 0.07 | 0.00 | 0.00 | 0.00 |
| 1A4bi | Domestic Closed Stove - Upgraded | Wood - Seasoned | 3.70 | 1.33 | 0.13 | 0.99 | 2.66 |
| 1A4bi | Domestic Fireplace - Standard | Coal | 1.85 | 1.19 | 0.32 | 1.08 | 2.16 |
| 1A4bi | Domestic Closed Stove - Upgraded | Wood - Wet | 1.73 | 1.33 | 0.13 | 0.99 | 2.66 |
| 1A4bi | Domestic Fireplace - Standard | Wood - Seasoned | 1.68 | 2.95 | 0.80 | 1.49 | 2.66 |
| 1A4bi | Domestic Closed Stove - Upgraded | Wood - Dry | 1.20 | 1.33 | 0.13 | 0.99 | 2.66 |
| 1A4bi | Domestic Fireplace - Standard | Wood - Wet | 0.78 | 2.95 | 0.80 | 1.49 | 2.66 |
| 1A4bi | Domestic Closed Stove - Basic | Wood - Seasoned | 0.74 | 2.66 | 0.13 | 0.99 | 2.66 |
| 1A4bi | Domestic Fireplace - Standard | Wood - Dry | 0.54 | 2.95 | 0.80 | 1.49 | 2.66 |
| 1A4bi | Domestic Outdoor | Charcoal | 0.40 | 0.93 | 0.32 | 1.08 | 2.16 |
| 1A4bi | Domestic Closed Stove - Basic | Wood - Wet | 0.35 | 2.66 | 0.13 | 0.99 | 2.66 |
| 1A4bi | Domestic Closed Stove - Basic | Wood - Dry | 0.24 | 2.66 | 0.13 | 0.99 | 2.66 |
| 1A4bi | Domestic Closed Stove - EcoDesign | Wood - Seasoned | 0.21 | 0.33 | 0.13 | 0.99 | 2.66 |
| 1A4bi | Domestic Water Heater | Natural gas | 0.19 | 0.01 | 0.00 | 0.00 | 0.00 |
| 1A4bi | Domestic Fireplace - Standard | SSF | 0.13 | 0.24 | 0.05 | 0.07 | 0.10 |
| 1A4bi | Domestic Closed Stove - Upgraded | Anthracite | 0.12 | 0.32 | 0.32 | 1.08 | 2.16 |
| 1A4ci | Agriculture - stationary combustion | Straw | 0.80 | 0.51 | 0.03 | 0.48 | 0.68 |
| 1A4cii | Agriculture - mobile machinery | Gas oil | 4.63 | 0.03 | 0.01 | 0.10 | 0.38 |
| 1A4ciii | Fishing vessels | Gas oil | 0.37 | 0.08 | 0.09 | 0.09 | 0.09 |
| 1A5b | Shipping - naval | Gas oil | 0.30 | 0.07 | 0.09 | 0.09 | 0.09 |

Table S6: Nitrogen Oxide (NO_x_) emission factors for 1A (energy industries) sources from the GAINS (low, medium, and high estimates) and NAEI emission factor databases that have greater than a 0.1% contribution to black carbon (BC) emissions (see table S1 caption).

|  | | | | **NOx EFs (kt/TWh)** | | | |
| --- | --- | --- | --- | --- | --- | --- | --- |
| **NFR Code** | **Source** | **Activity** | **Average contribution to total BC (%)** | **NAEI** | **GAINS Low** | **GAINS Medium** | **GAINS High** |
| 1A1a | Power stations | Wood | 0.15 | 0.25 | 0.13 | 0.30 | 0.60 |
| 1A1c | Upstream Oil Production - fuel combustion | Gas oil | 1.07 | 2.74 | 0.19 | 0.38 | 0.38 |
| 1A1c | Upstream Gas Production - fuel combustion | Gas oil | 0.26 | 3.73 | 0.19 | 0.38 | 0.38 |
| 1A2a | Iron and steel - combustion plant | Fuel oil | 0.61 | 0.36 | 0.14 | 0.35 | 0.70 |
| 1A2c | Chemicals (combustion) | Biomass | 0.17 | 0.35 | 0.36 | 0.36 | 0.72 |
| 1A2c | Chemicals (combustion) | Fuel oil | 0.15 | 0.36 | 0.14 | 0.35 | 0.70 |
| 1A2d | Pulp, Paper and Print (combustion) | Biomass | 3.42 | 0.35 | 0.36 | 0.36 | 0.72 |
| 1A2d | Pulp, Paper and Print (combustion) | Coal | 0.24 | 0.58 | 0.17 | 0.43 | 0.86 |
| 1A2e | Food & drink, tobacco (combustion) | Biomass | 0.50 | 0.35 | 0.36 | 0.36 | 0.72 |
| 1A2e | Food & drink, tobacco (combustion) | Coal | 0.16 | 0.58 | 0.17 | 0.43 | 0.86 |
| 1A2f | Mineral products (other): combustion | Biomass | 1.89 | 0.35 | 0.36 | 0.36 | 0.72 |
| 1A2gvii | Industrial off-road mobile machinery | Gas oil | 5.19 | 0.74 | 0.40 | 1.39 | 2.79 |
| 1A2gviii | Other industrial combustion | Biomass | 11.25 | 0.35 | 0.36 | 0.36 | 0.72 |
| 1A2gviii | Other industrial combustion | Burning oil | 5.22 | 1.85 | 0.14 | 0.35 | 0.70 |
| 1A2gviii | Other industrial combustion | Wood | 3.41 | 0.52 | 0.36 | 0.36 | 0.72 |
| 1A2gviii | Other industrial combustion | Coal | 1.56 | 0.58 | 0.17 | 0.43 | 0.86 |
| 1A2gviii | Other industrial combustion | Fuel oil | 0.82 | 0.80 | 0.14 | 0.35 | 0.70 |
| 1A2gviii | Other industrial combustion | Lubricants | 0.41 | 1.69 | 0.14 | 0.35 | 0.70 |
| 1A2gviii | Other industrial combustion | Gas oil | 0.26 | 1.69 | 0.14 | 0.35 | 0.70 |
| 1A2gviii | Other industrial combustion | Petroleum coke | 0.12 | 1.69 | 0.14 | 0.35 | 0.70 |
| 1A3c | Railways - intercity | Gas oil | 0.66 | 2.11 | 0.32 | 2.09 | 3.07 |
| 1A3c | Railways - regional | Gas oil | 0.60 | 1.57 | 0.32 | 2.09 | 3.07 |
| 1A3c | Railways - freight | Gas oil | 0.39 | 2.34 | 0.32 | 2.09 | 3.07 |
| 1A3dii | Shipping - coastal | Gas oil | 2.54 | 4.56 | 0.50 | 2.52 | 5.04 |
| 1A3dii | Motorboats / workboats | DERV | 1.96 | 3.56 | 0.40 | 2.09 | 4.64 |
| 1A3dii | Motorboats / workboats | Gas oil | 0.37 | 3.59 | 0.40 | 2.09 | 4.64 |
| 1A3dii | Shipping - coastal | Fuel oil | 0.33 | 6.11 | 0.50 | 2.52 | 5.04 |
| 1A3eii | Aircraft - support vehicles | Gas oil | 0.52 | 0.79 | 0.08 | 0.08 | 0.08 |
| 1A4ai | Misc. industrial/commercial combustion | Fuel oil | 0.16 | 1.10 | 0.14 | 0.35 | 0.70 |
| 1A4ai | Public sector combustion | Fuel oil | 0.14 | 0.36 | 0.14 | 0.35 | 0.70 |
| 1A4ai | Misc. industrial/commercial combustion | Gas oil | 0.11 | 1.52 | 0.14 | 0.27 | 0.27 |
| 1A4bi | Domestic Closed Stove - Upgraded | Wood - Seasoned | 3.70 | 0.23 | 0.25 | 0.25 | 0.25 |
| 1A4bi | Domestic Fireplace - Standard | Coal | 1.85 | 0.22 | 0.29 | 0.42 | 0.43 |
| 1A4bi | Domestic Closed Stove - Upgraded | Wood - Wet | 1.73 | 0.20 | 0.25 | 0.25 | 0.25 |
| 1A4bi | Domestic Fireplace - Standard | Wood - Seasoned | 1.68 | 0.16 | 0.25 | 0.25 | 0.25 |
| 1A4bi | Domestic Closed Stove - Upgraded | Wood - Dry | 1.20 | 0.14 | 0.25 | 0.25 | 0.25 |
| 1A4bi | Domestic Fireplace - Standard | Wood - Wet | 0.78 | 0.14 | 0.25 | 0.25 | 0.25 |
| 1A4bi | Domestic Closed Stove - Basic | Wood - Seasoned | 0.74 | 0.16 | 0.25 | 0.25 | 0.25 |
| 1A4bi | Domestic Fireplace - Standard | Wood - Dry | 0.54 | 0.16 | 0.25 | 0.25 | 0.25 |
| 1A4bi | Domestic Outdoor | Charcoal | 0.40 | 0.36 | 0.29 | 0.42 | 0.43 |
| 1A4bi | Domestic Closed Stove - Basic | Wood - Wet | 0.35 | 0.21 | 0.25 | 0.25 | 0.25 |
| 1A4bi | Domestic Closed Stove - Basic | Wood - Dry | 0.24 | 0.18 | 0.25 | 0.25 | 0.25 |
| 1A4bi | Domestic Closed Stove - EcoDesign | Wood - Seasoned | 0.21 | 0.18 | 0.25 | 0.25 | 0.25 |
| 1A4bi | Domestic Water Heater | Natural gas | 0.19 | 0.18 | 0.08 | 0.12 | 0.15 |
| 1A4bi | Domestic Fireplace - Standard | SSF | 0.13 | 0.22 | 0.29 | 0.42 | 0.43 |
| 1A4bi | Domestic Closed Stove - Upgraded | Anthracite | 0.12 | 0.36 | 0.29 | 0.42 | 0.43 |
| 1A4ci | Agriculture - stationary combustion | Straw | 0.80 | 0.33 | 0.25 | 0.25 | 0.60 |
| 1A4cii | Agriculture - mobile machinery | Gas oil | 4.63 | 0.62 | 0.27 | 1.78 | 3.96 |
| 1A4ciii | Fishing vessels | Gas oil | 0.37 | 5.58 | 0.47 | 2.34 | 4.68 |
| 1A5b | Shipping - naval | Gas oil | 0.30 | 5.86 | 0.50 | 2.52 | 5.04 |

Table S7: 2021 NAEI activity data used to produce NAEI BC emissions estimates and applied to GAINS BC EF datasets.

| **NFR** | **Source** | **Fuel** | **TWh** |
| --- | --- | --- | --- |
| 1A1a | Heat supply | Landfill gas | 0.143721 |
| 1A1a | Heat supply | Sewage gas | 0.920853 |
| 1A1a | Miscellaneous industrial/commercial combustion | MSW | 2.66976 |
| 1A1a | Power stations | Coal | 17.34671 |
| 1A1a | Power stations | Coke | 0 |
| 1A1a | Power stations | Landfill gas | 8.374248 |
| 1A1a | Power stations | LPG | 0 |
| 1A1a | Power stations | MSW | 26.23063 |
| 1A1a | Power stations | Natural gas | 206.3742 |
| 1A1a | Power stations | OPG | 0 |
| 1A1a | Power stations | Orimulsion | 0 |
| 1A1a | Power stations | Scrap tyres | 0 |
| 1A1a | Power stations | Sewage gas | 2.463051 |
| 1A1a | Power stations | Slurry | 0 |
| 1A1b | Refineries - combustion | Burning oil | 0 |
| 1A1b | Refineries - combustion | Fuel oil | 1.268806 |
| 1A1b | Refineries - combustion | Gas oil | 0 |
| 1A1b | Refineries - combustion | LPG | 0.053743 |
| 1A1b | Refineries - combustion | Naphtha | 0.012364 |
| 1A1b | Refineries - combustion | Natural gas | 11.02726 |
| 1A1b | Refineries - combustion | OPG | 25.36878 |
| 1A1b | Refineries - combustion | Petrol | 0 |
| 1A1b | Refineries - combustion | Refinery miscellaneous | 0 |
| 1A1c | Coke production | Blast furnace gas | 0.527759 |
| 1A1c | Coke production | Coke oven gas | 1.005444 |
| 1A1c | Coke production | Colliery methane | 0 |
| 1A1c | Coke production | Natural gas | 0 |
| 1A1c | Collieries - combustion | Coal | 0 |
| 1A1c | Collieries - combustion | Coke oven gas | 0 |
| 1A1c | Collieries - combustion | Colliery methane | 0.267768 |
| 1A1c | Collieries - combustion | Natural gas | 0.054174 |
| 1A1c | Gas production | Colliery methane | 0 |
| 1A1c | Gas production | LPG | 0 |
| 1A1c | Gas production | Natural gas | 5.501919 |
| 1A1c | Gas production | OPG | 0 |
| 1A1c | Gas production | Town gas | 0 |
| 1A1c | Gas terminal: fuel combustion | Gas oil | 0.007467 |
| 1A1c | Gas terminal: fuel combustion | Natural gas | 5.87009 |
| 1A1c | Nuclear fuel production | Natural gas | 0 |
| 1A1c | Oil terminal: fuel combustion | Gas oil | 0.214009 |
| 1A1c | Oil terminal: fuel combustion | Natural gas | 2.783595 |
| 1A1c | Solid smokeless fuel production | Coke | 0 |
| 1A1c | Solid smokeless fuel production | Natural gas | 0 |
| 1A1c | Town gas manufacture | Burning oil | 0 |
| 1A1c | Town gas manufacture | Coal | 0 |
| 1A1c | Town gas manufacture | Coke | 0 |
| 1A1c | Town gas manufacture | Coke oven gas | 0 |
| 1A1c | Town gas manufacture | LPG | 0 |
| 1A1c | Town gas manufacture | Natural gas | 0 |
| 1A1c | Upstream Gas Production - fuel combustion | Gas oil | 0.77491 |
| 1A1c | Upstream Gas Production - fuel combustion | Natural gas | 7.497845 |
| 1A1c | Upstream oil and gas production - combustion at gas separation plant | LPG | 0 |
| 1A1c | Upstream oil and gas production - combustion at gas separation plant | OPG | 0 |
| 1A1c | Upstream Oil Production - fuel combustion | Gas oil | 5.304505 |
| 1A1c | Upstream Oil Production - fuel combustion | Natural gas | 24.50942 |
| 1A2a | Iron and steel - combustion plant | Biogas | 0 |
| 1A2a | Iron and steel - combustion plant | Biomass | 0 |
| 1A2a | Iron and steel - combustion plant | Coal | 0.165819 |
| 1A2a | Iron and steel - combustion plant | Coke | 0.036422 |
| 1A2a | Iron and steel - combustion plant | Fuel oil | 0.265609 |
| 1A2a | Iron and steel - combustion plant | Gas oil | 0.040474 |
| 1A2a | Iron and steel - combustion plant | LPG | 0.004723 |
| 1A2a | Iron and steel - combustion plant | Natural gas | 4.993609 |
| 1A2a | Iron and steel - combustion plant | Town gas | 0 |
| 1A2b | Autogeneration - exported to grid | Coal | 0.06021 |
| 1A2b | Autogenerators | Coal | 0.06804 |
| 1A2b | Non-Ferrous Metal (combustion) | Biogas | 0 |
| 1A2b | Non-Ferrous Metal (combustion) | Biomass | 0 |
| 1A2b | Non-Ferrous Metal (combustion) | Coal | 0.191054 |
| 1A2b | Non-Ferrous Metal (combustion) | Fuel oil | 0.003637 |
| 1A2b | Non-Ferrous Metal (combustion) | Gas oil | 0 |
| 1A2b | Non-Ferrous Metal (combustion) | Natural gas | 2.8986 |
| 1A2c | Chemicals (combustion) | Biogas | 0.043497 |
| 1A2c | Chemicals (combustion) | Biomass | 0.352026 |
| 1A2c | Chemicals (combustion) | Coal | 0.35329 |
| 1A2c | Chemicals (combustion) | Fuel oil | 0.15853 |
| 1A2c | Chemicals (combustion) | Gas oil | 0 |
| 1A2c | Chemicals (combustion) | Natural gas | 24.32022 |
| 1A2d | Pulp, Paper and Print (combustion) | Biogas | 0.143192 |
| 1A2d | Pulp, Paper and Print (combustion) | Biomass | 2.617622 |
| 1A2d | Pulp, Paper and Print (combustion) | Coal | 0.722803 |
| 1A2d | Pulp, Paper and Print (combustion) | Fuel oil | 0.008069 |
| 1A2d | Pulp, Paper and Print (combustion) | Gas oil | 0 |
| 1A2d | Pulp, Paper and Print (combustion) | Natural gas | 5.21307 |
| 1A2e | Food & drink, tobacco (combustion) | Biogas | 0.124142 |
| 1A2e | Food & drink, tobacco (combustion) | Biomass | 0.640172 |
| 1A2e | Food & drink, tobacco (combustion) | Coal | 0.423784 |
| 1A2e | Food & drink, tobacco (combustion) | Fuel oil | 0.026592 |
| 1A2e | Food & drink, tobacco (combustion) | Gas oil | 0 |
| 1A2e | Food & drink, tobacco (combustion) | Natural gas | 19.5229 |
| 1A2f | Lime production - non decarbonising | Coke | 0 |
| 1A2f | Mineral products (other): combustion | Biogas | 0 |
| 1A2f | Mineral products (other): combustion | Biomass | 2.762536 |
| 1A2gvii | Industrial off-road mobile machinery | DERV | 0 |
| 1A2gvii | Industrial off-road mobile machinery | Gas oil | 18.22135 |
| 1A2gvii | Industrial off-road mobile machinery | Petrol | 3.278995 |
| 1A2gviii | Autogeneration - exported to grid | Natural gas | 8.407549 |
| 1A2gviii | Autogenerators | Biogas | 9.390033 |
| 1A2gviii | Autogenerators | Natural gas | 9.780682 |
| 1A2gviii | Construction (combustion) | Biogas | 0 |
| 1A2gviii | Construction (combustion) | Biomass | 0 |
| 1A2gviii | Electrical engineering (combustion) | Biogas | 0 |
| 1A2gviii | Electrical engineering (combustion) | Biomass | 0 |
| 1A2gviii | Mechanical Engineering (combustion) | Biogas | 0 |
| 1A2gviii | Mechanical Engineering (combustion) | Biomass | 0 |
| 1A2gviii | Other industrial combustion | Biogas | 0 |
| 1A2gviii | Other industrial combustion | Biomass | 19.90362 |
| 1A2gviii | Other industrial combustion | Burning oil | 18.92548 |
| 1A2gviii | Other industrial combustion | Coal | 3.849175 |
| 1A2gviii | Other industrial combustion | Coke oven gas | 0.001251 |
| 1A2gviii | Other industrial combustion | Colliery methane | 0.003681 |
| 1A2gviii | Other industrial combustion | Fuel oil | 1.320274 |
| 1A2gviii | Other industrial combustion | Gas oil | 0.812393 |
| 1A2gviii | Other industrial combustion | LPG | 10.17456 |
| 1A2gviii | Other industrial combustion | Natural gas | 46.47329 |
| 1A2gviii | Other industrial combustion | SSF | 0 |
| 1A2gviii | Other industrial combustion | Town gas | 0 |
| 1A2gviii | Other industrial combustion | Wood | 3.336812 |
| 1A2gviii | Textiles and leather (combustion) | Biogas | 0 |
| 1A2gviii | Textiles and leather (combustion) | Biomass | 0 |
| 1A3ai(i) | Aircraft between UK and Bermuda - TOL | Aviation spirit | 0 |
| 1A3ai(i) | Aircraft between UK and Bermuda - TOL | Aviation turbine fuel | 0.004109 |
| 1A3ai(i) | Aircraft between UK and CDs - TOL | Aviation spirit | 0 |
| 1A3ai(i) | Aircraft between UK and CDs - TOL | Aviation turbine fuel | 0.052434 |
| 1A3ai(i) | Aircraft between UK and Gibraltar - TOL | Aviation spirit | 0 |
| 1A3ai(i) | Aircraft between UK and Gibraltar - TOL | Aviation turbine fuel | 0.018815 |
| 1A3ai(i) | Aircraft between UK and other OTs (excl Gib. and Bermuda) - TOL | Aviation spirit | 0 |
| 1A3ai(i) | Aircraft between UK and other OTs (excl Gib. and Bermuda) - TOL | Aviation turbine fuel | 0.000775 |
| 1A3aii(i) | Aircraft - domestic take off and landing | Aviation spirit | 0.13294 |
| 1A3aii(i) | Aircraft - domestic take off and landing | Aviation turbine fuel | 0.722706 |
| 1A3bi | Road transport - all vehicles LPG use | LPG | 0.755332 |
| 1A3bi | Road transport - cars - cold start | DERV | 102.8986 |
| 1A3bi | Road transport - cars - cold start | Petrol | 115.7316 |
| 1A3bi | Road transport - cars - motorway driving | DERV | 18.25093 |
| 1A3bi | Road transport - cars - motorway driving | Petrol | 17.34145 |
| 1A3bi | Road transport - cars - rural driving | DERV | 45.13635 |
| 1A3bi | Road transport - cars - rural driving | Petrol | 44.73847 |
| 1A3bi | Road transport - cars - urban driving | DERV | 39.57912 |
| 1A3bi | Road transport - cars - urban driving | Petrol | 53.72916 |
| 1A3bii | Road transport - all vehicles biofuels use | Biodiesel | 11.37719 |
| 1A3bii | Road transport - all vehicles biofuels use | Biogas | 0 |
| 1A3bii | Road transport - all vehicles biofuels use | Biomass | 0 |
| 1A3bii | Road transport - all vehicles biofuels use | Bio-MTBE | 0.007703 |
| 1A3bii | Road transport - LGVs - cold start | DERV | 66.41429 |
| 1A3bii | Road transport - LGVs - cold start | Petrol | 1.913598 |
| 1A3bii | Road transport - LGVs - motorway driving | DERV | 13.88799 |
| 1A3bii | Road transport - LGVs - motorway driving | Petrol | 0.362086 |
| 1A3bii | Road transport - LGVs - rural driving | DERV | 29.72426 |
| 1A3bii | Road transport - LGVs - rural driving | Petrol | 0.834386 |
| 1A3bii | Road transport - LGVs - urban driving | DERV | 22.83203 |
| 1A3bii | Road transport - LGVs - urban driving | Petrol | 0.717963 |
| 1A3biii | Road transport - buses and coaches - motorway driving | DERV | 0.352323 |
| 1A3biii | Road transport - buses and coaches - rural driving | DERV | 2.415799 |
| 1A3biii | Road transport - buses and coaches - urban driving | DERV | 5.899775 |
| 1A3biii | Road transport - general | Natural gas | 0.88257 |
| 1A3biii | Road transport - HGV articulated - motorway driving | DERV | 28.10707 |
| 1A3biii | Road transport - HGV articulated - rural driving | DERV | 19.40079 |
| 1A3biii | Road transport - HGV articulated - urban driving | DERV | 4.152022 |
| 1A3biii | Road transport - HGV rigid - motorway driving | DERV | 8.112408 |
| 1A3biii | Road transport - HGV rigid - rural driving | DERV | 12.84689 |
| 1A3biii | Road transport - HGV rigid - urban driving | DERV | 7.092677 |
| 1A3biv | Road transport - mopeds (<50cc 2st) - urban driving | Lubricants | 0.001379 |
| 1A3biv | Road transport - mopeds (<50cc 2st) - urban driving | Petrol | 0.022695 |
| 1A3biv | Road transport - motorcycle (>50cc 2st) - urban driving | Petrol | 0.013109 |
| 1A3biv | Road transport - motorcycle (>50cc 4st) - motorway driving | Petrol | 0.104568 |
| 1A3biv | Road transport - motorcycle (>50cc 4st) - rural driving | Petrol | 0.593229 |
| 1A3biv | Road transport - motorcycle (>50cc 4st) - urban driving | Petrol | 1.112902 |
| 1A3c | Rail - coal | Coal | 0.111028 |
| 1A3c | Railways - freight | Gas oil | 1.600763 |
| 1A3c | Railways - intercity | Gas oil | 1.8776 |
| 1A3c | Railways - regional | Gas oil | 2.166345 |
| 1A3dii | Inland goods-carrying vessels | DERV | 0 |
| 1A3dii | Inland goods-carrying vessels | Gas oil | 0.020088 |
| 1A3dii | Inland goods-carrying vessels | Petrol | 0 |
| 1A3dii | Motorboats / workboats (e.g. canal boats, dredgers, service boats, tourist boats, river boats) | DERV | 1.588672 |
| 1A3dii | Motorboats / workboats (e.g. canal boats, dredgers, service boats, tourist boats, river boats) | Gas oil | 0.258026 |
| 1A3dii | Motorboats / workboats (e.g. canal boats, dredgers, service boats, tourist boats, river boats) | Petrol | 0.88505 |
| 1A3dii | Personal watercraft e.g. jet ski | DERV | 0 |
| 1A3dii | Personal watercraft e.g. jet ski | Gas oil | 0 |
| 1A3dii | Personal watercraft e.g. jet ski | Petrol | 0.666583 |
| 1A3dii | Sailing boats with auxiliary engines | DERV | 0.034134 |
| 1A3dii | Sailing boats with auxiliary engines | Gas oil | 0 |
| 1A3dii | Sailing boats with auxiliary engines | Petrol | 0 |
| 1A3dii | Shipping - coastal | Fuel oil | 1.786878 |
| 1A3dii | Shipping - coastal | Gas oil | 12.49278 |
| 1A3dii | Shipping between UK and Bermuda | Fuel oil | 5.99E-05 |
| 1A3dii | Shipping between UK and CDs | Fuel oil | 0.002894 |
| 1A3dii | Shipping between UK and CDs | Gas oil | 0.062318 |
| 1A3dii | Shipping between UK and Gibraltar | Fuel oil | 0.133577 |
| 1A3dii | Shipping between UK and OTs (excl. Gib and Bermuda) | Fuel oil | 0.001445 |
| 1A3eii | Aircraft - support vehicles | Gas oil | 0.480607 |
| 1A4ai | Miscellaneous industrial/commercial combustion | Biogas | 0 |
| 1A4ai | Miscellaneous industrial/commercial combustion | Biomass | 0 |
| 1A4ai | Miscellaneous industrial/commercial combustion | Coal | 0.088192 |
| 1A4ai | Miscellaneous industrial/commercial combustion | Coke | 0 |
| 1A4ai | Miscellaneous industrial/commercial combustion | Fuel oil | 0.249897 |
| 1A4ai | Miscellaneous industrial/commercial combustion | Gas oil | 0.447579 |
| 1A4ai | Miscellaneous industrial/commercial combustion | LPG | 0.119583 |
| 1A4ai | Miscellaneous industrial/commercial combustion | Natural gas | 54.86536 |
| 1A4ai | Miscellaneous industrial/commercial combustion | SSF | 0 |
| 1A4ai | Miscellaneous industrial/commercial combustion | Town gas | 0 |
| 1A4ai | Public sector combustion | Biogas | 0 |
| 1A4ai | Public sector combustion | Biomass | 0.021574 |
| 1A4ai | Public sector combustion | Burning oil | 0 |
| 1A4ai | Public sector combustion | Coal | 0.173725 |
| 1A4ai | Public sector combustion | Coke | 0 |
| 1A4ai | Public sector combustion | Fuel oil | 0.112732 |
| 1A4ai | Public sector combustion | Gas oil | 0.28006 |
| 1A4ai | Public sector combustion | Natural gas | 36.22083 |
| 1A4ai | Public sector combustion | Town gas | 0 |
| 1A4ai | Railways - stationary combustion | Burning oil | 0 |
| 1A4ai | Railways - stationary combustion | Coal | 0 |
| 1A4ai | Railways - stationary combustion | Coke | 0 |
| 1A4ai | Railways - stationary combustion | Fuel oil | 0 |
| 1A4ai | Railways - stationary combustion | Natural gas | 0.014262 |
| 1A4aii | Industrial off-road mobile machinery | LPG | 0.751011 |
| 1A4bi | Domestic Boiler | Anthracite | 0.752111 |
| 1A4bi | Domestic Boiler | Coal | 0.191339 |
| 1A4bi | Domestic Boiler | Coke | 0 |
| 1A4bi | Domestic Boiler | LPG | 2.622946 |
| 1A4bi | Domestic Boiler | Petroleum coke | 0.192258 |
| 1A4bi | Domestic Boiler | SSF | 0.323805 |
| 1A4bi | Domestic Closed Stove - Basic | Anthracite | 0.081448 |
| 1A4bi | Domestic Closed Stove - Basic | Coal | 0.014271 |
| 1A4bi | Domestic Closed Stove - Basic | Coke | 0 |
| 1A4bi | Domestic Closed Stove - Basic | Petroleum coke | 0.031527 |
| 1A4bi | Domestic Closed Stove - Basic | SSF | 0.053098 |
| 1A4bi | Domestic Closed Stove - Basic | Wood - Dry | 0.123662 |
| 1A4bi | Domestic Closed Stove - Basic | Wood - Seasoned | 0.382124 |
| 1A4bi | Domestic Closed Stove - Basic | Wood - Wet | 0.178481 |
| 1A4bi | Domestic Closed Stove - EcoDesign | Anthracite | 0.151047 |
| 1A4bi | Domestic Closed Stove - EcoDesign | Coal | 0.026466 |
| 1A4bi | Domestic Closed Stove - EcoDesign | Coke | 0 |
| 1A4bi | Domestic Closed Stove - EcoDesign | Petroleum coke | 0.058467 |
| 1A4bi | Domestic Closed Stove - EcoDesign | SSF | 0.098472 |
| 1A4bi | Domestic Closed Stove - EcoDesign | Wood - Dry | 0.229335 |
| 1A4bi | Domestic Closed Stove - EcoDesign | Wood - Seasoned | 0.70866 |
| 1A4bi | Domestic Closed Stove - EcoDesign | Wood - Wet | 0.330998 |
| 1A4bi | Domestic Closed Stove - Upgraded | Anthracite | 0.587953 |
| 1A4bi | Domestic Closed Stove - Upgraded | Coal | 0.10302 |
| 1A4bi | Domestic Closed Stove - Upgraded | Coke | 0 |
| 1A4bi | Domestic Closed Stove - Upgraded | Petroleum coke | 0.227585 |
| 1A4bi | Domestic Closed Stove - Upgraded | SSF | 0.383303 |
| 1A4bi | Domestic Closed Stove - Upgraded | Wood - Dry | 0.892688 |
| 1A4bi | Domestic Closed Stove - Upgraded | Wood - Seasoned | 2.758469 |
| 1A4bi | Domestic Closed Stove - Upgraded | Wood - Wet | 1.288414 |
| 1A4bi | Domestic combustion | Charcoal | 0.000352 |
| 1A4bi | Domestic combustion | Town gas | 0 |
| 1A4bi | Domestic Cooking and Other | Burning oil | 0 |
| 1A4bi | Domestic Cooking and Other | Fuel oil | 0 |
| 1A4bi | Domestic Cooking and Other | Gas oil | 0 |
| 1A4bi | Domestic Cooking and Other | Natural gas | 7.04423 |
| 1A4bi | Domestic Fireplace - Standard | Coal | 2.099615 |
| 1A4bi | Domestic Fireplace - Standard | Coke | 0 |
| 1A4bi | Domestic Fireplace - Standard | Peat | 0.012664 |
| 1A4bi | Domestic Fireplace - Standard | Petroleum coke | 0.371076 |
| 1A4bi | Domestic Fireplace - Standard | SSF | 0.624976 |
| 1A4bi | Domestic Fireplace - Standard | Wood - Dry | 0.504282 |
| 1A4bi | Domestic Fireplace - Standard | Wood - Seasoned | 1.558265 |
| 1A4bi | Domestic Fireplace - Standard | Wood - Wet | 0.727828 |
| 1A4bi | Domestic Outdoor | Charcoal | 0.822943 |
| 1A4bi | Domestic Outdoor | Coal | 1.96E-05 |
| 1A4bi | Domestic Space Heater | Burning oil | 18.37035 |
| 1A4bi | Domestic Space Heater | Fuel oil | 0 |
| 1A4bi | Domestic Space Heater | Gas oil | 1.015066 |
| 1A4bi | Domestic Space Heater | Natural gas | 215.1591 |
| 1A4bi | Domestic Water Heater | Burning oil | 4.212887 |
| 1A4bi | Domestic Water Heater | Fuel oil | 0 |
| 1A4bi | Domestic Water Heater | Gas oil | 0.232786 |
| 1A4bi | Domestic Water Heater | Natural gas | 65.12964 |
| 1A4bii | House and garden machinery | DERV | 0.129757 |
| 1A4bii | House and garden machinery | Petrol | 1.373704 |
| 1A4ci | Agriculture - stationary combustion | Biogas | 0.075247 |
| 1A4ci | Agriculture - stationary combustion | Biomass | 0 |
| 1A4ci | Agriculture - stationary combustion | Burning oil | 0 |
| 1A4ci | Agriculture - stationary combustion | Coal | 0 |
| 1A4ci | Agriculture - stationary combustion | Coke | 0 |
| 1A4ci | Agriculture - stationary combustion | Fuel oil | 0.061366 |
| 1A4ci | Agriculture - stationary combustion | Gas oil | 0.010568 |
| 1A4ci | Agriculture - stationary combustion | Natural gas | 1.241231 |
| 1A4ci | Agriculture - stationary combustion | Straw | 0.850746 |
| 1A4ci | Agriculture - stationary combustion | Vaporising oil | 0 |
| 1A4cii | Agriculture - mobile machinery | Gas oil | 17.77264 |
| 1A4cii | Agriculture - mobile machinery | Petrol | 0 |
| 1A4ciii | Fishing vessels | Fuel oil | 0.092383 |
| 1A4ciii | Fishing vessels | Gas oil | 1.630467 |
| 1A5b | Aircraft - military | Aviation spirit | 0.030831 |
| 1A5b | Aircraft - military | Aviation turbine fuel | 4.00572 |
| 1A5b | Shipping - naval | Gas oil | 1.940603 |

**NAEI BC Emissions and NFR Scoping**

Figure S1: Number of BC emissions sources within 1A with an average contribution above 0.1% (averaged across the years 2015 – 2021). In total, 83 sources had an average above 0.1%.


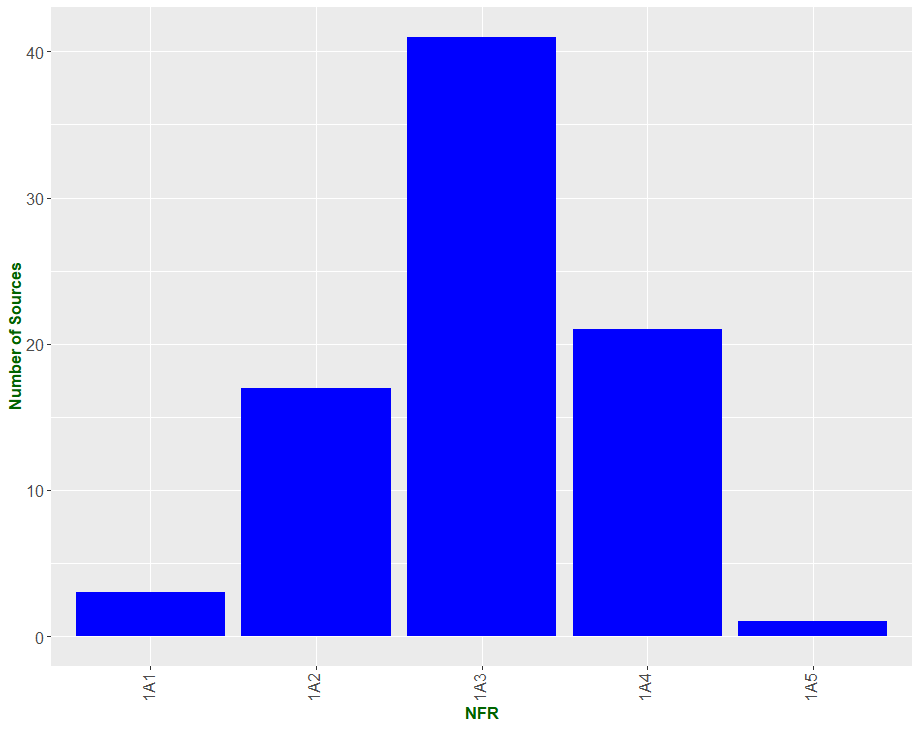

Supplement: Supplementary file 1 — Supplementary Material 1: The following supplementary information can be found at:. Table S1: Black carbon emission factors from the NAEI and GAINS datasets. Table S2: Non-exhaust black carbon and PM2.5 road transport emission factors from the GAINS and NAEI (EMEP/EEA) databases. Table S3: Exhaust-based black carbon, PM2.5, and NOx road transport emission factors from the GAINS and NAEI (EMEP/EEA) databases. Table S4: NAEI exhaust-based road transport contributions to total annual BC emissions, by source. Table S5: PM2.5 emission factors from the NAEI and GAINS datasets. Table S6: NOx emission factors from the NAEI and GAINS datasets. Table S7: 2021 NAEI activity data for BC emissions estimates. Figure S1: Number of sources within each NAEI 1 A NFR code (sub-sector) with an annual average contribution to BC emissions above 0.1%. [file 13021_2025_306_MOESM1_ESM.docx]
